# Photocross-Linkable and Shape-Memory Biomaterial Hydrogel Based on Methacrylated Cellulose Nanofibres

**DOI:** 10.1021/acs.biomac.3c00476

**Published:** 2023-08-01

**Authors:** Yury Brusentsev, Peiru Yang, Alistair W. T. King, Fang Cheng, Maria F. Cortes Ruiz, John E. Eriksson, Ilkka Kilpeläinen, Stefan Willför, Chunlin Xu, Lars Wågberg, Xiaoju Wang

**Affiliations:** † Laboratory of Natural Materials Technology, Johan Gadolin Process Chemistry Centre, Åbo Akademi University, Henrikinkatu 2, 20500 Turku, Finland; ‡ Turku Bioscience Centre, University of Turku and Åbo Akademi University, Tykistökatu 6, 20520 Turku, Finland; § Cell Biology, Faculty of Science and Engineering, Åbo Akademi University, Tykistökatu 6, 20520 Turku, Finland; ∥ Chemistry Department, University of Helsinki, Yliopistonkatu 3, 00014 Helsinki, Finland; ⊥ School of Pharmaceutical Sciences (Shenzhen), Shenzhen Campus of Sun Yat-sen University, Shenzhen 518107, China; # Department of Fibre and Polymer Technology, Division of Fibre Technology, KTH Royal Institute of Technology, Teknikringen 56-58, 100 44 Stockholm, Sweden; ∇ Department of Fibre and Polymer Technology, Wallenberg Wood Science Centre, KTH Royal Institute of Technology, Teknikringen 56-58, 100 44 Stockholm, Sweden

## Abstract

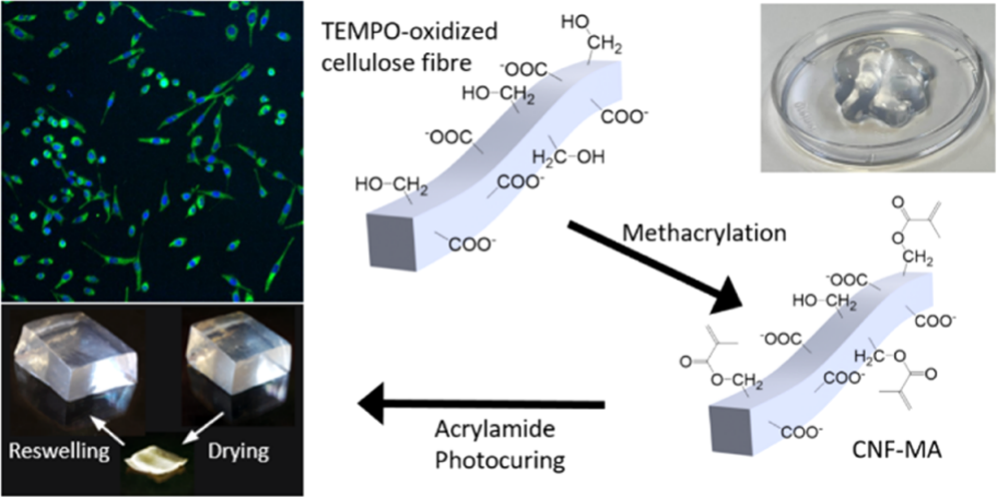

In the context of three-dimensional (3D) cell culture and tissue engineering, 3D printing is a powerful tool for customizing in vitro 3D cell culture models that are critical for understanding the cell–matrix and cell–cell interactions. Cellulose nanofibril (CNF) hydrogels are emerging in constructing scaffolds able to imitate tissue in a microenvironment. A direct modification of the methacryloyl (MA) group onto CNF is an appealing approach to synthesize photocross-linkable building blocks in formulating CNF-based bioinks for light-assisted 3D printing; however, it faces the challenge of the low efficiency of heterogenous surface modification. Here, a multistep approach yields CNF methacrylate (CNF-MA) with a decent degree of substitution while maintaining a highly dispersible CNF hydrogel, and CNF-MA is further formulated and copolymerized with monomeric acrylamide (AA) to form a super transparent hydrogel with tuneable mechanical strength (compression modulus, approximately 5–15 kPa). The resulting photocurable hydrogel shows good printability in direct ink writing and good cytocompatibility with HeLa and human dermal fibroblast cell lines. Moreover, the hydrogel reswells in water and expands to all directions to restore its original dimension after being air-dried, with further enhanced mechanical properties, for example, Young’s modulus of a 1.1% CNF-MA/1% PAA hydrogel after reswelling in water increases to 10.3 kPa from 5.5 kPa.

## Introduction

In a very up-to-date research context of tissue engineering, extrusion-based three-dimensional (3D) bioprinting is largely applied as a tool for building in vitro 3D cell culture models or tissue equivalents in order to understand the underlying cell–matrix and cell–cell interactions in, e.g., either cancer models or tissue regeneration.^1,2^ In the field of biomaterials, the fast development of bioinks of different kinds is currently being unconstrainedly explored.^3,4^ Technically, the most striving bioink materials are expected to offer excellent ink fidelity and workability in terms of filament resolution, construct integrity, and geometry complexity of the hydrogel constructs. Meanwhile, cellular compatibility of the biomaterials is a primary request for the selection of formulation components in order for their bioinks to support the adhesion, proliferation, and differentiation of live cells.^5^ What is more important, extracellular matrix (ECM)-mimicking microenvironments are necessary for the formation of relevant tissue models for embedding cells. Hence, the hydrogel matrix in a bioink that is formed via either physiochemical interactions or covalent bonds to form an interpenetrating network of macromolecules should provide a multimodal control over the material properties such as surface nanomorphology, viscoelasticity/stiffness, or delivery functions of biochemical factors.^6^ For the formulation of bioinks, a wide spectrum of natural polymer-based biomaterials are available from the bioactive ECM-components, e.g., collagen, fibronectin, and hyaluronic acid, to more abundant natural polysaccharides, e.g., chitosan, alginate, and ulvan.^7−10^ More practically, this catalog has been extended to their semisynthetic methacrylated derivatives, such as methacrylated gelatine (GelMA) and methacrylated hyaluronic acid (HEMA), in the endeavor to consolidate the printing fidelity with the external auxiliary process of modest cross-linking and to tune the hydrogel properties in the printing process as well as in the form of printed constructs.^11^

In recent years, cellulosic nanofibril (CNF) hydrogels have emerged as a highly functional non-ECM component in formulating bioinks,^12^ both in research and in newly commercialized bioink formulations. Typically, CNFs are produced by mechanical disintegration from a sustainable resource of wood pulp, with or without pre-treatment of different types (e.g., enzymatic treatment by, for example, cellulase, TEMPO oxidation, periodate oxidation, or phosphorylation) to result in cellulose fibrils with diameters of 5–15 nm and lengths of approximately up to a micrometer.^13^ At a comparatively low-concentration range of 1–3 wt % (mainly dependent on the surface chemistry of the CNFs), these flexible nanofibrils with a high aspect ratio are physically entangled to form a translucent hydrogel with a stable microstructure and excellent viscoelastic properties.^14,15^ Also, CNF hydrogels, without covalent cross-links and with monovalent ions as counterions, display shear-thinning rheology and can flow upon shear by disrupting the fibril entanglement.^16^ Moreover, the nanofibrils possess a fibrous ECM-mimicking morphology that actually supports cell–matrix interactions and has paved the way for the success of CNF hydrogels as a generic 3D cell culture matrix.^17^ As previously reported, the CNF hydrogel also stands out in the ability to form low-concentration ink systems (less than 5 wt % dry content), ascribed to its reinforcing effect when formulated with a secondary biopolymer such as alginate, gelatine, or wood polysaccharides.^18,19^ Excellent ink properties of these CNF-based bioinks have been achieved with different cross-linking strategies, such as physical cross-linking of alginate by Ca^2+^,^14^ enzymatic cross-linking of the tyramine-modified xylan^20^ by horseradish peroxidase, and photocross-linking of GelMA^19^ or methacrylated galactoglucomannan.^21^

Light-assisted 3D bioprinting with bioinks containing methacrylated biopolymers has become prevailingly popular as the photochemical-activated free radical chain polymerization of methacryloyl groups is cytocompatible and allows the synthesis of versatile hydrogels with tuneable mechanical properties.^22,23^ In this scenario, a direct modification of the methacryloyl (MA) group onto cellulose nanofibrils is an appealing approach to synthesize photocross-linkable building blocks in formulating CNF-based bioinks. Very recently, Ma et al.^24^ reported the synthesis of methacrylated CNF (CNF-MA) by reacting the CNF suspended from dry powder in water with methacrylic anhydride and further utilized the methacrylated CNF to reinforce a UV-curable soy protein resin in direct molding and laser 3D printing. The surface methacrylation on CNF materials was not directly proven by NMR, and a quantitative analysis on the substitution degree (DS) of MA groups was not available to reflect the efficiency of the product in photocross-linking. In fact, the surface modification of the CNF materials in water is challenging as the synthesis is requested to induce efficient surface modification while preserving the water-swollen status of the surface-modified nanofibrils in the hydrogels. Chemical modification of never-dried cellulose in aqueous medium via alkoxysilane chemistry has also been a successful approach by avoiding the irreversible hornification processes in drying.^25^ Elegant examples of reacting the CNF surface with functionalized triethoxysilane under acidic conditions lead to formation of functional groups such as vinyl, amino, and azido functionalities onto the cellulose surface.^26^ Another approach is with 1-ethyl-3-(3-dimethylaminopropyl) carbodiimide (EDC)/*N*-hydroxysuccinimide (NHS)-assisted amidation to the carboxyl groups on the TEMPO-oxidized CNF.^27^ With this method, the degree of amidation shall be taken into careful consideration to avoid the aggregation of nanofibrils when part of the carboxylic (−COO^–^) groups are converted to amide groups.

With our endeavor to develop the methacrylated CNF-based photocross-linkable ink formulation, a decent DS of MA on the cellulose nanofibrils is necessary while maintaining a highly dispersible CNF hydrogel that can be applicable in 3D printing. Here, we present a multistep procedure, including (i) TEMPO-mediated oxidation of a fully bleached kraft pulp in aqueous medium, (ii) solvent exchange of the oxidized cellulose fibers from water to dimethylformamide (DMF) avoiding fiber wall collapse, i.e., maintaining an open fiber wall structure, (iii) surface modification of hydroxy groups on the fiber with methacrylic anhydride in DMF, and (iv) complete removal of DMF and mechanical defibrillation of the methacrylated cellulose fibers in water, to obtain free fibrils that will form the desired dispersible CNF-MA hydrogel at low concentrations. Furthermore, to obtain a precise quantification of the DS in CNF-MA, we adapted the system of tetra-*n*-butyl-phosphonium acetate (*n*-Bu_4_P^+^OAc^–^) in DMSO-*d*_6_ as a solvent in solution-state NMR for the as-synthesized CNF-MA, as earlier developed by King et al.^28^ Next, the CNF-MA was copolymerized with monomeric acrylamide (AA) as a feedstock ink for hydrogel extrusion-based 3D printing, as polyacrylamide, the polymerization product of AA, is generally accepted to be noncytotoxic in biomedical applications. The photocross-linking kinetics of CNF-MA with monomeric AA was studied via photorheology. The cytotoxicity of the photocross-linked CNF-MA+AA hydrogel was assessed in the culture of HeLa cancer cell line and human dermal fibroblasts (HDFs). Lastly, the printability of the CNF-MA+AA ink formulation was also preliminarily evaluated in direct ink writing (DIW).

## Experimental Section

### Materials

All chemicals and solvents used for the chemical modification and for cross-linking of cellulose were purchased from Sigma-Aldrich or VWR Chemicals if not otherwise mentioned. Solvent for the NMR analysis of the cellulose materials was prepared according to the method and from the materials described in a previous publication.^28^

### Preparation of Oxidized CNF with TEMPO

TEMPO-oxidized CNF was prepared from Spruce Kraft Pulp (produced by Borregaard, Norway) by the method reported by Liu et al.^29^ The oxidized fibers with a charge density of 1.25 ± 0.03 mmol/g were used for subsequent modification.

### Preparation of CNF-MA

128 g of the 7.8% TEMPO-oxidized fiber in water (1.25 mmol/g charge, 10 g dry mass) was pressed on a filter to remove water. Then, the fiber mat was dispersed in 300 mL of DMF and filtered to remove most of the solvents, followed by washing with 150 mL of DMF three times. The washed oxidized fibers were redispersed in 300 mL of DMF, to which, 10 mL (73 mmol) of triethylamine was added. Afterward, 8.5 mL (56 mmol) of methacrylic anhydride was added dropwise for 15 min. The reaction mixture was stirred overnight with light protection. The thus-modified fibers were filtered and washed with water (300 mL, 5 times) and then concentrated to approximately 10% of the dry weight. The fiber dispersion in water was stored in cold with light protection until homogenization prior to further use.

### Charge Density

The charge was determined for the prepared fiber by conductometric titration following the protocol reported previously.^30^ CNF-MA charge was found to be 1.07 ± 0.02 mmol/g. The prepared fibers were analyzed by NMR spectroscopy.

### NMR Measurement

NMR spectra were recorded with Bruker Advance 400 and 500 MHz NMR spectrometers using standard pulse sequences. After the removal of sodium ions (see details in the Supporting Information), the CNF-MA was dissolved in the electrolyte containing 20% of *n*-Bu_4_P^+^OAc^–^ and 80% of DMSO-*d*_6_. The solution was characterized by diffusion-filtered ^1^H NMR using methacrylic group signals compared to the C1-H signal of the glucose or glucuronic acid unit of cellulose for quantification of the DS of modification. It determined the amount of the methacrylic groups of 2.0 mol % glucose or glucuronic acid unit of the cellulose material, which corresponded to DS 0.02 or ≈0.12 mmol/g.

### Atomic Force Microscopy (AFM) Measurement

AFM imaging was performed for the visualization of the individual fibrils of CNF-MA and nonmodified oxidized (n/m-) CNF. For the imaging, a Bruker MultiMode 8 AFM with an NCHV-A probe was utilized. Imaging was performed in tapping mode with a tip radius of around 10 nm. The surfaces were prepared on clean silicon wafers. The CNFs were adsorbed by first adsorbing polyethylenimine (PEI, 0.1 g/L) followed by corresponding CNF at a concentration of 0.1 g/L.

### Transmission Electron Microscopy (TEM) Measurement

TEM images were acquired by a JEOL transmission electron microscope. TEM images were captured for diluted solutions (0.01 g/L) of CNF-MA and n/m-CNF. Staining was performed with a 0.1% uranyl acetate solution. The thickness of the fibrils and fibril length were determined from the images. Values of 3–5 nm for thickness and 300–1600 nm for the length of the fibrils are in good agreement with AFM and dynamic light scattering (DLS) results.

### Brunauer–Emmett–Teller (BET) Measurement

BET surface area analysis was performed using a Micromeritics 3flex instrument with a VacPrep061 degasser prior to the nitrogen adsorption measurement. Two aerogel samples for the BET nitrogen sorption analysis were prepared from 1.1 wt % CNF-MA and 1.1 wt % n/m-CNF hydrogel materials cross-linked by CaCl_2_ solution. Water in the samples was gradually changed to ethanol followed by carbon dioxide (CO_2_) critical point drying to preserve the original structure of CNF. Critical point drying was performed by an Autosamdri-815 instrument with 20 min liquid CO_2_ purging time. The samples were degassed for 12 h at 60 °C before the BET measurement. The analysis resulted in surface areas of 456 m^2^/g for the CNF-MA aerogel and 441 m^2^/g for the n/m-CNF material. Pore volumes were 0.20 cm^3^/g and 0.19 cm^3^/g for CNF-MA and n/m-CNF materials, respectively.

### Photorheology

Rheology studies were carried out using a TA Discovery HR-2 rheometer with a UV LED Curing Pate and 20 mm smooth parallel plate geometry. The rheological experiment was used to evaluate changes in mechanical properties, which take place in the hydrogel under UV curing. Time sweep oscillation of 5 min along with a constant strain of 1% and a shear rate of 1 s^–1^ was used. UV (power varied from 15 to 32.5 mW/cm^2^) was switched on after 50 s of oscillation. With this method (UV power of 15 mW/cm^2^), the first three formulations were evaluated including 1.1% CNF-MA, 0.2% Irgacure D-2959; 1.1% n/m-CNF, 1% acrylamide, 0.4% Irgacure D-2959; and 1.1% CNF-MA, 1% acrylamide, 0.4% Irgacure D-2959. The optimization of the concentration of initiator Irgacure D-2959 was performed with gels composed of CNF-MA 1.1%, acrylamide 1%, and Irgacure D-2959 0–0.4% (UV power of 15 mW/cm^2^). The optimization of the acrylamide concentration presented was performed with gels composed of CNF-MA 1.1%, acrylamide 0.25–4%, and Irgacure D-2959 0.2% (UV power of 15 mW/cm^2^).

### Compression Test

Compression tests were performed on a TA Discovery HR-2 rheometer with a Peltier Plate and 25 mm flat geometry. Samples for the tests were prepared from the formulation containing 1.1% CNF, 1% acrylamide, and 0.2% initiator Irgacure 2959 (Ink-1). The cylindrical mold (9.6 mm inner diameter, 50 mm length) was filled with the formulation. The filled mold was cross-linked by UV irradiation (Cellink Inkredable 3D printer-integrated UV LED 365 nm, 2 cm from the source) for 5 min. A prepared long cylinder with a diameter of 9.6 mm was cut by a razor into small cylinders with heights of 6–8 mm. Three samples were air-dried for 24 h (21 °C, 25% relative humidity) and reswelled for 2 h (the same diameter formed). A few samples were soaked in phosphate-buffered saline (PBS) for 24 h (shrank to a diameter of 8.9 mm). For reference, the Ink-1 formulation (10 mL) was cross-linked with by addition of 0.1 mL of 5% CaCl_2_ solution in the mold (after 48 h, it was cut into similar samples). The compression test was performed using a rheometer with a compression speed of 500 μm/min. The compression force profiles were converted to pressure profiles using appropriate sample cross-sectional areas (*P* = *F*/*a*; *a* = π*d*^2^/4, *d* = 9.6 mm for all of the samples besides the PBS soaked where *d* = 8.9 mm). Young’s modulus was determined as the slope of the linear approximation of the compression curve in the initial linear part (0–10% compression for all of the samples besides 0–4% for air-dried/reswelled samples; *E* = *P*/σ). More samples were prepared for the compression analyses using the same procedure. Materials containing CNF-MA 1.1%, Irgacure D-2959 0.2%, and acrylamide with concentrations 0.25, 0.5, 1, 1.5, 2, and 4% were used for cross-linking in the cylindrical molds. After cutting into small cylinders, the samples were tested as described above. Young’s modulus values were calculated for the samples with different acrylamide concentrations.

### SEM Measurement

The morphology of the surfaces was characterized by SEM imaging using a Leo 1530 Gemini instrument. The aerogel materials for the SEM imaging were prepared from the UV-cross-linked “Ink-1” formulation by gradually changing the solvent from water to ethanol, followed by carbon dioxide critical point drying. The procedure of the aerogel preparation was performed to preserve the original structure of the hydrogel. The comparison sample was prepared from the formulation of 1.1% n/m-CNF, 1% acrylamide, and 0.2% Irgacure 2959. Acrylamide in the sample gel was polymerized by UV and then the gel was cross-linked by addition of a 5% CaCl_2_ solution. The material was treated the same way as the first sample to get the aerogel. The aerogels were sputter-coated with platinum prior to imaging.

### Swelling Behavior

To investigate the prepared material (“Ink-1”—1.1% CNF, 1% acrylamide, and 0.2% initiator Irgacure 2959, cross-linked with UV) for the swelling ability, two sets of samples were prepared. Cross-linked gel cylinders were prepared as described for the compression tests. Four samples were left for 24 h for air-drying at room temperature conditions (22 °C, 25% relative humidity). The weight of the residue was 2.5% (average of four samples) of the original gel (dry content 2.3%, the difference of ∼10% could be residual moisture content). Three samples were freeze-dried for 18 h to give 2.48% (average of three samples) of the original gel (dry content 2.3%, the difference of ∼8% could again be residual moisture and an error of the concentration in the prepared gel). The samples were placed then into deionized water and the kinetics of water uptake was determined. To investigate how the time of drying affects the swelling behavior, two samples were prepared from the Ink-1 formulation and cross-linked by UV curing. The samples were air-dried for 24 and 120 h (22 °C, 25% relative humidity) to give shapeless residues of 2.7 and 2.5% of the original gels, respectively. To investigate the effect of the water residue on the swelling ability, two samples of the cross-linked Ink-1 material were air-dried for 24 h and then vacuum-dried at high vacuum and room temperature for 20 h. All the samples were placed in water for swelling. The water uptake was registered for 2 and 24 h of swelling.

### Cell Culture

HDFs and HeLa cells were used for the cytocompatibility evaluation of CNF-MA hydrogels. Both cell lines were maintained in 10 cm Petri dishes in a humid CO_2_ incubator with a preset temperature of 37 °C. The cell medium was changed every 2 days with Dulbecco’s modified Eagle high glucose medium (DMEM, high glucose) supplemented with 2 mM l-glutamine, 100 IU/mL penicillin/streptomycin(P/S), and 10% heat-inactivated fetal bovine serum (FBS). Cells were passaged upon reaching 70% percent confluency.

### Hydrogel Coating

CNF-MA (1.1%) hydrogels containing 0.25, 0.5, or 1% AA and 0.2% Irgacure D-2959 were dispensed in 96-well plates and 24-well plates, respectively. The hydrogel precursors were cross-linked by a UV LED (365 nm, 15 mW/cm^2^, bluepoint LED eco, Hönle Group) for 1 min to form a flat hydrogel coating on the well bottom. The hydrogel coating was further sterilized under UV (254 nm) for 30 min. Then, the coated plates were washed with sterile Dulbecco’s phosphate-buffered saline (DPBS) 3–5 times to remove potential free acrylamide monomers and were stored in a +4 °C fridge until further experiments. Hydrogel-coated plates were preincubated with supplemented DMEM overnight in a humidified CO_2_ incubator before plating cells.

### Cell Proliferation Assay and Cytotoxicity Assay

100 μL of HDFs and HeLa cell suspensions were dispensed separately in 96-well plates (5000 cells/well) precoated with different CNF-MA hydrogels as mentioned above. Seeded plates were incubated in a CO_2_ incubator at 37 °C for 1, 2, 3, 5, and 7 days. The cell culture medium was changed regularly every 2 days. At the end of each time point, 10 μL of CCK-8 solution (Donjito) was added to each testing well of the plates. Absorbance was measured at 450 nm using a microplate reader after 1 h incubation at 37 °C.

### Immunofluorescent Staining and Confocal Microscopy

HDF and HeLa cells were seeded in 24-well glass-bottom plates precoated with different CNF-MA hydrogels as mentioned above. Cells were fixed with 4% paraformaldehyde for 15 min and stained with phalloidin (Ex. 647 nm) and 4′,6-diamidino-2-phenylindole (DAPI, Ex. 405 nm) subsequently. The cells were mounted with PBS with 0.03% NaN_3_ in plates. *Z*-stack images were taken using confocal microscopy (3i CSU-W1 spinning disk, 10× Zeiss Plan-Apochromat, NA = 0.8). Images were processed with ImageJ, where maximal projection was applied to show maximal intensity.

### DIW Printing

The 3D printability and shape fidelity of the developed formulations were evaluated by an extrusion-based 3D printer INKREDIBLE+ (CELLINK) with a pneumatic dispensing system and a cylindrical 250 μm stainless steel nozzle. 0.85–1.3% CNF-MA/1% polyacrylamide inks containing 0.2% Irgacure 2959 as the photoinitiator were extruded from a 5 mL syringe to determine the printability of CNF hydrogels. 1.1% CNF-MA was found to be the optimal concentration. Different pressures (10–24 kPa) were used to optimize the extrusion speed. 14 kPa was found to be the optimal extrusion pressure for 1.1% CNF-MA inks. Three-dimensional printing using 1.1% CNF-MA/1% polyacrylamide inks containing 0.2% Irgacure 2959 as the photoinitiator was performed. After the whole object was printed, post-UV cross-linking with a printer-integrated UV light source for 10 min was applied. To check the printability and strut resolution, the inks were tested with the printing of 1–5 mm mesh and scaffold constructs in the dimensions of 10 mm × 10 mm and a height of 2 mm. To determine the maximum number of layers deposited on each other without fusing, circular grids of the 1.5 mm mesh with 6–12 layers were printed. No significant fusing was observed in up to 10 layers of constructs. To check the shape fidelity of intricate objects, the ear geometry (40 mm × 25 mm × 5 mm) was printed. All of the printing works were conducted with the same parameters including a printing speed of 240 mm/min and an extruding pressure of 14 kPa.

## Results and Discussion

### Methacrylated Cellulose Nanofibres (CNF-MA): Their Synthesis, Balance between Surface Charge and DS of Methacrylates, and Their Fibril Morphology

**Figure 1:**
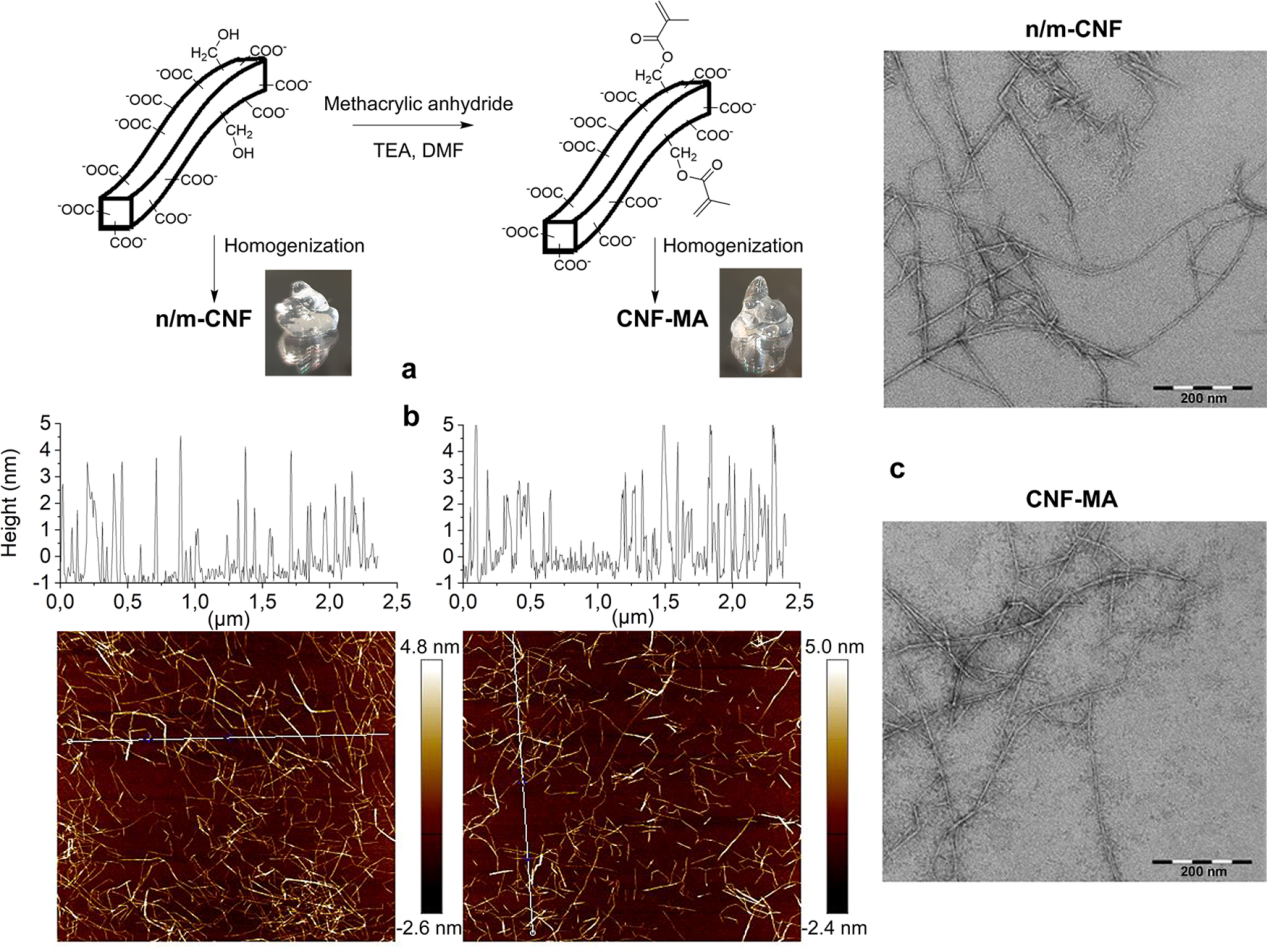
(a) Illustration of the surface modification of fibers with TEMPO oxidation (top left) and methacrylic anhydride (top right) in DMF, both of which yielded highly transparent viscous hydrogels as shown in the photos. (b) AFM characterization of fibrils of n/m-CNF (left) and CNF-MA (right) with line profile analysis (on the top), showing that the numerical values of fibril heights remain consistent after MA modification. (c) TEM images of n/m-CNF and CNF-MA. Abbreviations: n/m-CNF: nonmodified oxidized CNF and CNF-MA: methacrylic modified oxidized CNF.

**Figure 2:**
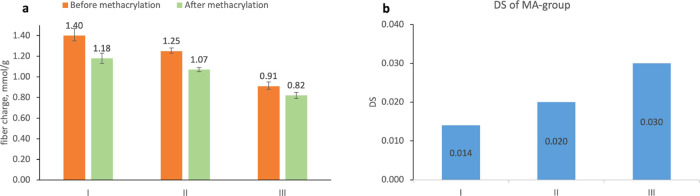
(a) Fibril charges before and after methacrylation. (b) Modification degree of the MA group. The detailed assignment of methacrylic functional groups in the modified products (CNF-MA) and the comparison of regular and diffusion-filtered ^1^H NMR spectra to obtain reliable DS values are reported in the Supporting Information (Figure S1a–d).

In the DIW printing of a nanocellulose hydrogel, a binary ink formulated with a cross-linkable component is often used to ensure good ink performance in terms of shape fidelity and tailored mechanical properties of the printed ink.^19,21^ Photocross-linkable polymers, e.g., different polymers of methacrylates or methacrylated natural polymers, are favored due to the high efficiency and easy-to-apply processing properties, as well as high printing resolution of the prepared objects.^22^ However, grafting from polymerization of nanocellulose fibrils, which takes advantage of using fibrils as “backbones,” is still sparingly reported. Here, CNF-MA was synthesized to enable in situ polymerization of the MA moiety anchored on the fibril surface. TEMPO-mediated oxidation was used to open up the fiber wall and to separate the fibrils from each other and hence activate the fibril surface prior to methacrylation (Figure 1a) and this resulted in cellulose fibrils with charge densities of 0.91, 1.25, and 1.40 mmol/g (Figure 2). Afterward, water was exchanged for DMF in order to increase the reaction efficiency for further derivatization with methacrylic anhydride. The oxidized fibrils with a charge density of 0.91 mmol/g showed the highest DS for MA, 0.03. A higher charge density had a negative impact on the DS for MA. This can most probably be explained by the lower availability of hydroxyl groups in the oxidized fibrils with the higher content of −COO^–^ groups. It is also observed that the amount of the −COO^–^ groups after MA derivatization has decreased, which is most probably ascribed to the hydrolysis of the cellulose that might have occurred during solvent exchange from water to DMF, derivatization, and back to water again. The oxidized and methacrylated fibers were homogenized in water suspension to produce cellulose nanofibrils (n/m-CNF—nonmodified oxidized CNF and CNF-MA—methacrylic modified oxidized CNF).

Modification of the cellulose fibril surface, especially in an organic solvent, often alters the structural properties of fibrils.^31^ Thus, the modified fibrils were thoroughly characterized to assess their morphological and chemical properties. AFM imaging of the individual fibrils of the modified and nonmodified CNF (Figure 1b) showed similar dimensions, with a width in the range from 3 to 5 nm for both samples. The same fibril width range could also be observed by TEM imaging (Figure 1c). The fiber length could be determined to be in the range from 200 nm to a couple of micrometers. Dynamic light scattering (DLS) measurement confirmed that methacrylation did not lead to aggregation or sedimentation of the fibrils (Table S1). Furthermore, the specific surface areas of the aerogels prepared from both n/m-CNF and CNF-MA hydrogels with supercritical CO_2_ drying were measured using the BET nitrogen adsorption method. Both materials showed very similar surface areas (420 m^2^/g for nonmodified and 450 m^2^/g for CNF-MA) and similar pore size distributions (Table S2).

### UV Cross-Linkable Hydrogels Formed by Copolymerization of CNF-MA and Acrylamide (AA): Photochemistry Kinetics, Mechanical Characteristics, and Swelling Behavior of Hydrogels of CNF-MA+AA

**Figure 3:**
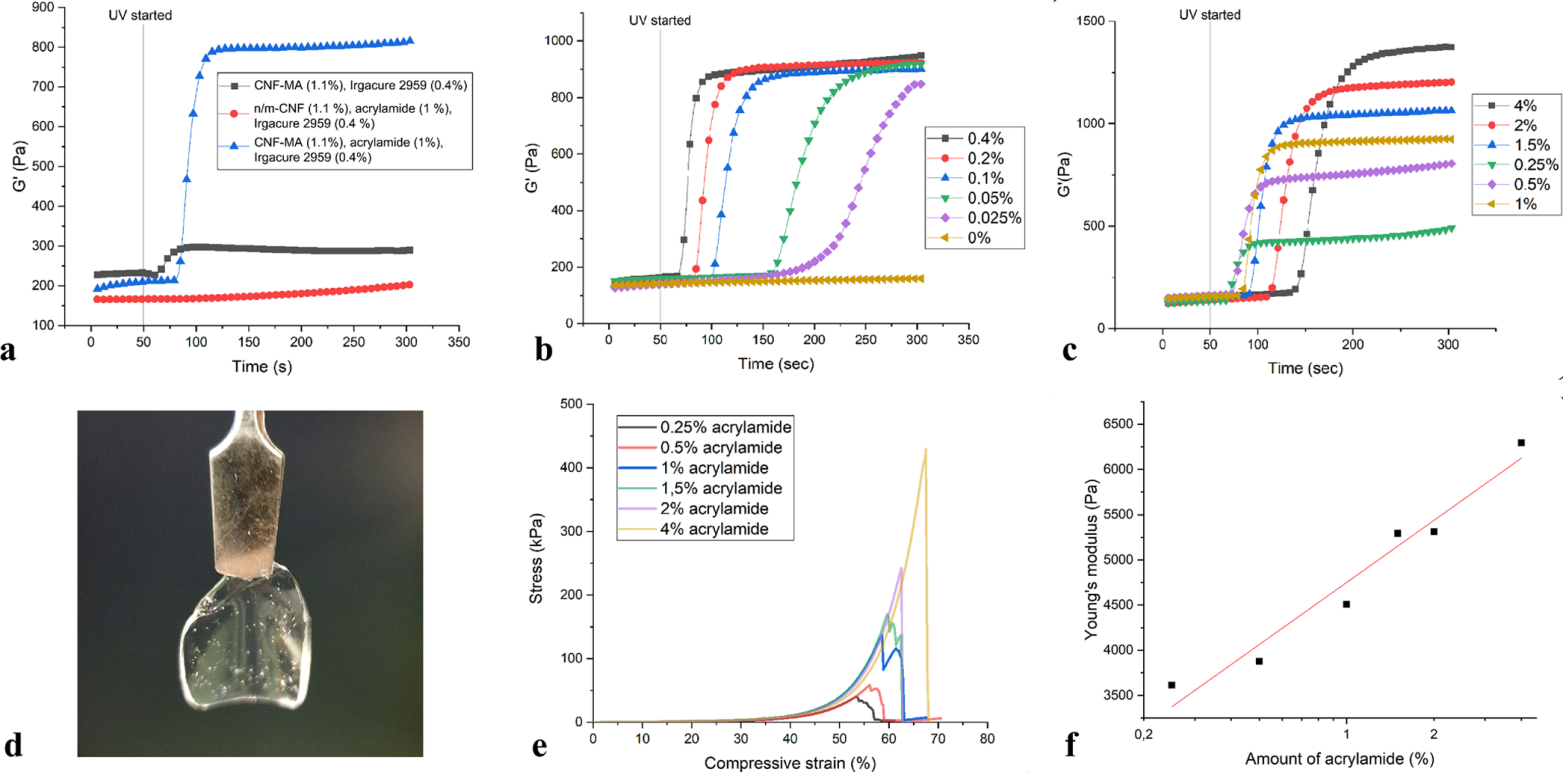
(a) Photorheology time sweeps of hydrogels with three formulations: 1. CNF-MA (1.1%), Irgacure 2959 initiator (0.4%) only; 2. n/m-CNF (1.1%), acrylamide monomer (1%), Irgacure 2959 (0.4%); 3. CNF-MA (1.1%), acrylamide (1%), Irgacure 2959 (0.4%). (b) Photorheology time sweeps of hydrogels of CNF-MA (1.1%), acrylamide (1%), and Irgacure 2959 with varying additions (0–0.4%). (c) Photorheology time sweeps of hydrogels of CNF-MA (1.1%), Irgacure 2959 (0.2%), and acrylamide with varying additions (0.25–4%). (d) Photocured disc as a result of the photorheology experiment (CNF-MA (1.1%), acrylamide (1%), and Irgacure 2959 (0.4%); UV 365 nm for 20 min). (e) Compression curves for the cross-linked hydrogels containing CNF-MA (1.1%), Irgacure 2959 (0.2%), and acrylamide with varying additions (0.25–4%). (f) Linear approximation of Young’s modulus (average value) as a function of the logarithm of acrylamide concentration for hydrogels containing CNF-MA (1.1%), Irgacure 2959 (0.2%), and acrylamide with varying additions (0.25–4%).

In our endeavor to synthesize photoreactive CNF-based hydrogels, the MA-modified CNFs (CNF-MA) were copolymerized with acrylamide under UV irradiation at 365 nm. Acrylamide was chosen due to the accessibility and good biomechanical properties of the polyacrylamide gels in water.^32^ CNF-MA with 1 mmol/g surface charge and 0.02 methacrylate modification degree was selected as an adequate level of surface ionization is essential to maintain the good colloidal stability of the CNFs in order to have a good distribution of the fibrils in the final material. It should be emphasized, though, that an intermediate level of surface charge of −COO^–^ has been shown to better support the proliferative activity of cell lines, such as fibroblasts.^33^ For optimization of the final materials, these two factors naturally have to be balanced. To determine the photochemistry reaction kinetics, the storage modulus, *G*′, was continuously monitored during UV irradiation in the rheometer at a constant strain of 1% and a frequency of 1 s^–1^, as shown in Figure 3a. The Irgacure 2959 photoinitiator concentration was initially set at 0.4%, and the *G*′ of CNF-MA (1.1%), with only the photoinitiator and no acrylamide, showed a very weak enhancement upon the UV exposure, and no acceptable hydrogel with a high enough integrity could be collected under these conditions. These results indicate that there is a weak cross-linking of methacrylate groups in the pristine CNF-MA but that it is not sufficient to support a self-sustained network. As expected, the addition of 1% acrylamide into 1.1% CNF-MA showed a rapid and profound *G*′ increase upon UV irradiation, which in turn resulted in an elastic hydrogel with good integrity, as also shown in Figure 3a,d. As a control to test the influence of MA grafting to the CNF, the *G*′ of the 1% n/m-CNF+1% acrylamide formulation remained unchanged under UV exposure (Figure 3a), which indeed showed that the copolymerization of CNF-MA and acrylamide accounted for the formation of a continuous fibrillar network linked together by polyacrylamide chains covalently attached to the fibrils. To estimate the length of the fibril–fibril linkages, the acrylamide cross-linked CNF-MA gel was prepared (1.1% CNF-MA, 1% acrylamide, 0.4% Irgacure 2959, UV 365 nm for 20 min). It was then extracted with water to remove nonbonded polyacrylamide and then subjected to basic hydrolysis to cleave the binding polymer. After extraction and purification, the binding polymer was analyzed for molecular weight with HP-SEC and for chemical composition by NMR. The average *M*_W_ of the polymer was determined as 300 kDa (polydispersity 1.6), and the ratio of the acrylamide to the methacrylic unit in the polymer was around 100:1. The amount of polyacrylamide grafted to CNF-MA fibrils was calculated to accounting for approximately 70% (see the Determination of the Length of the Cross-Linker PAA section in the Supporting Information for details,). A coarse estimation based on these results, together with the assumption that the chain growth occurs along the fibril, gives the maximum length of the linker of around 2500 acrylamide units or roughly 1.5 μm assuming a fully extended polymer chain (see Figures S3 and S4 in the Supporting Information for the details). If the linker polymer binds several fibrils, the length between fibrils could naturally be multiple times less.

In order to optimize the photoinitiator concentration, the photochemistry kinetics of the 1.1% CNF-MA+1% acrylamide formulation was established at different concentrations of Irgacure 2959 from 0 to 0.4% using the in situ rheology polymerization technique. The responses of *G*′ upon UV irradiation are shown in Figure 3b. In general, a higher photoinitiator concentration resulted in faster UV curing. Only when the concentration of Irgacure 2959 was above 0.1%, a complete degree of cross-linking (i.e., *G*′ reaching a plateau) can be achieved within a UV exposure duration of less than 30 s. Rapid photocross-linking is critical in UV-aided DIW printing to support good printing fidelity. Based on these results, addition of 0.2% Irgacure 2959 was selected as optimal for further use, since a too high concentration of Irgacure 2959 might have a risk of causing cell toxicity.

The acrylamide cross-linker content was also optimized using the photorheology tool with the CNF-MA content set as 1.1% and with the alteration of the acrylamide content from 0.25 to 4%. The responses of their *G*′ upon UV exposure are presented in Figure 3c. The results show that the addition of acrylamide increased the gelation time, as a longer time was required to form enough radicals when the concentration of the initiator was kept the same. At the same time, a larger content of acrylamide resulted in a higher value of *G*′ for the UV-cross-linked hydrogel of CNF-MA+acrylamide, which shows increased elastic behavior at higher monomer concentrations that might be ascribed to the higher cross-linking rate or reinforcing effect of unbound polymers. Still, the UV-cross-linked hydrogels of CNF-MA+PAA turned out to be relatively soft materials with *G*′ values lower than 1.5 kPa. In addition to the rheological evaluation, the mechanical response of this group of UV-cross-linked CNF-MA+PAA hydrogels was measured by an axial compression test, and the results are shown as stress–strain curves in Figure 3e. The CNF-MA+PAA hydrogels possessed remarkable mechanical properties, showing good elasticity with a fracture strain >55% when the monomeric acrylamide content was above 1.0%. In Figure 3f, Young’s modulus derived from the stress–strain curves is displayed for all four repetitive samples in each set of acrylamide contents of 0.25, 0.5, 1.0, 2.0, or 4.0%. Young’s modulus showed a linear dependence on the logarithm of acrylamide content in the formulations, and it can be concluded that a higher cross-linking density creates a stiffer network, which is expected. The UV-cross-linked 1.1% CNF-MA+4% PAA hydrogel showed the highest compressive stress of 0.45 MPa at a compressive strain of 68%.

**Figure 4:**
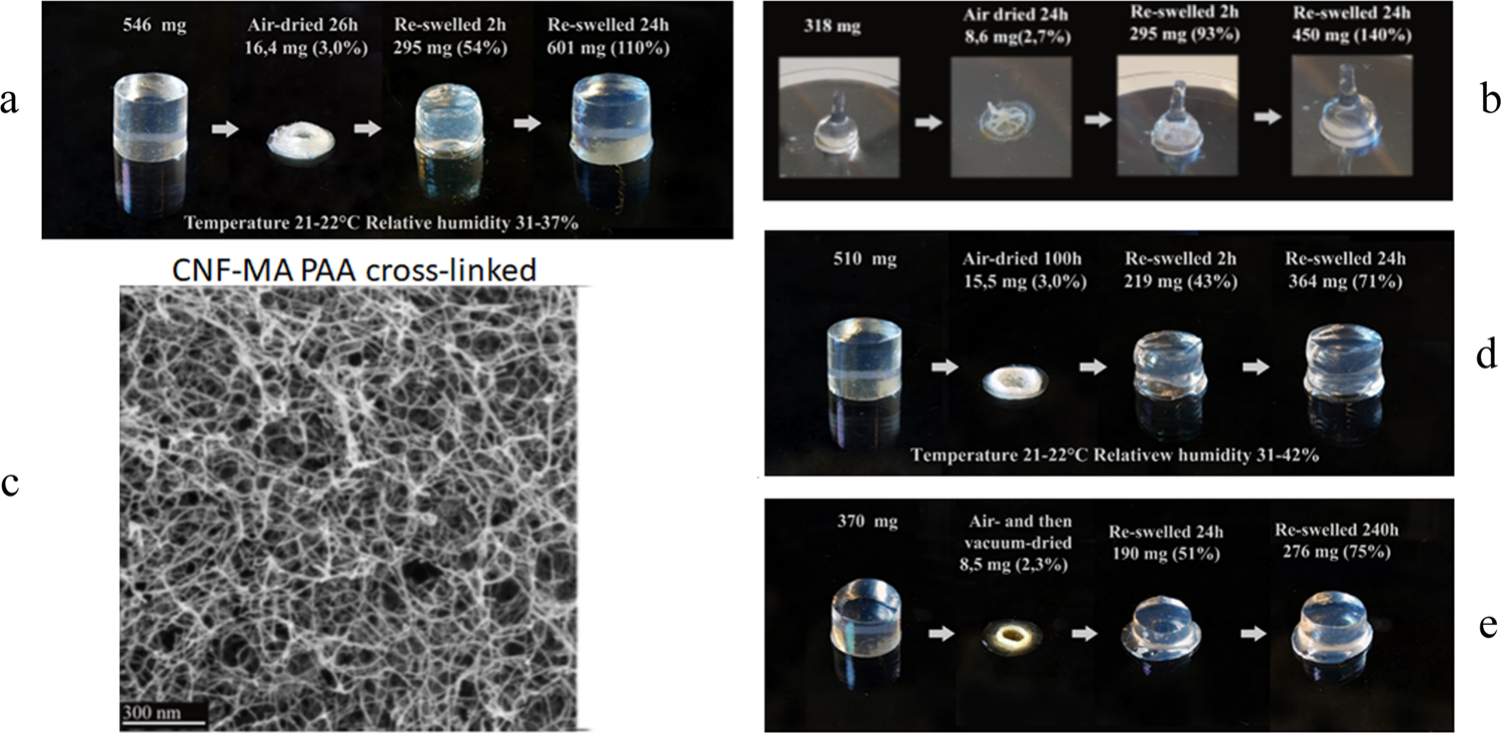
Swelling behavior of aerogels of the photocross-linked CNF-MA+AA hydrogel via different drying procedures (a. air-dried for 26 h and reswelled in 24 h; b. air-dried for 24 h and reswelled in 24 h; d. air-dried for 100 h and reswelled in 24 h; and e. air- and vacuum-dried and reswelled in 240 h) and the SEM image (c) of the cross sections of the CNF-MA cross-linked with PAA.

In targeted applications such as a biomaterial matrix for supporting a 3D cell culture, it would be an excellent approach to be able to dry the CNF-based hydrogels into films/aerogels and to reswell them in an applicable medium for storage and transportation of active components. In order to evaluate the properties of the UV-cross-linked hydrogels (1.1% CNF-MA+1% acrylamide), the reswelling behavior after different drying conditions was evaluated. As illustrated in Figure 4a, a cylinder-shaped hydrogel was air-dried for 24 h to arrive at a dry film, and then the film was allowed to reswell for 24 h in deionized water, which actually restored its original geometry. The excellent shape-memory effect of the 1.1% CNF-MA+1% PAA hydrogel (upon 24 h air-drying) was also demonstrated with a hydrogel “teat” with an even more complex geometry, as shown in Figure 4b. These swelling properties are quite different from what has been observed for the drying of CNF gels, where the charges were kept in the Na^+^ form.^34^ For these gels, the drying resulted in a maintained diameter of the gels, followed by subsequent swelling only in the *z*-direction. For the covalently cross-linked systems in the present work, the drying and reswelling of the gels seem to occur in all directions. The reason for this is that the fibrillar network of the gel is covalently cross-linked, and when the water is removed, the gel shrinks in all directions, and upon reswelling, it expands in all directions. The maintained open structure of the covalently cross-linked network is shown in the SEM images of the gels subjected to supercritical CO_2_ drying in Figure 4c and this is a prerequisite for the excellent properties of the covalently cross-linked system.

However, a more extended air-drying time (100 h) and vacuum-drying decreased the rate of water uptake during reswelling and, to some, minor, extent, limited the gel’s ability to restore its original shape, as demonstrated for the samples displayed in Figure 4d,e with the reswelling kinetics presented and discussed more comprehensively in the Supporting Information (Figures S5–S7). Under these drying conditions, it is suggested that the well-known irreversible association of cellulose surfaces during heating and drying, also known as hornification,^35,36^ being more profound when the drying process was extended, resulted in lower porosity and poorer solvent accessibility and hence deteriorated the reswelling capability of the dry films.

**Figure 5:**
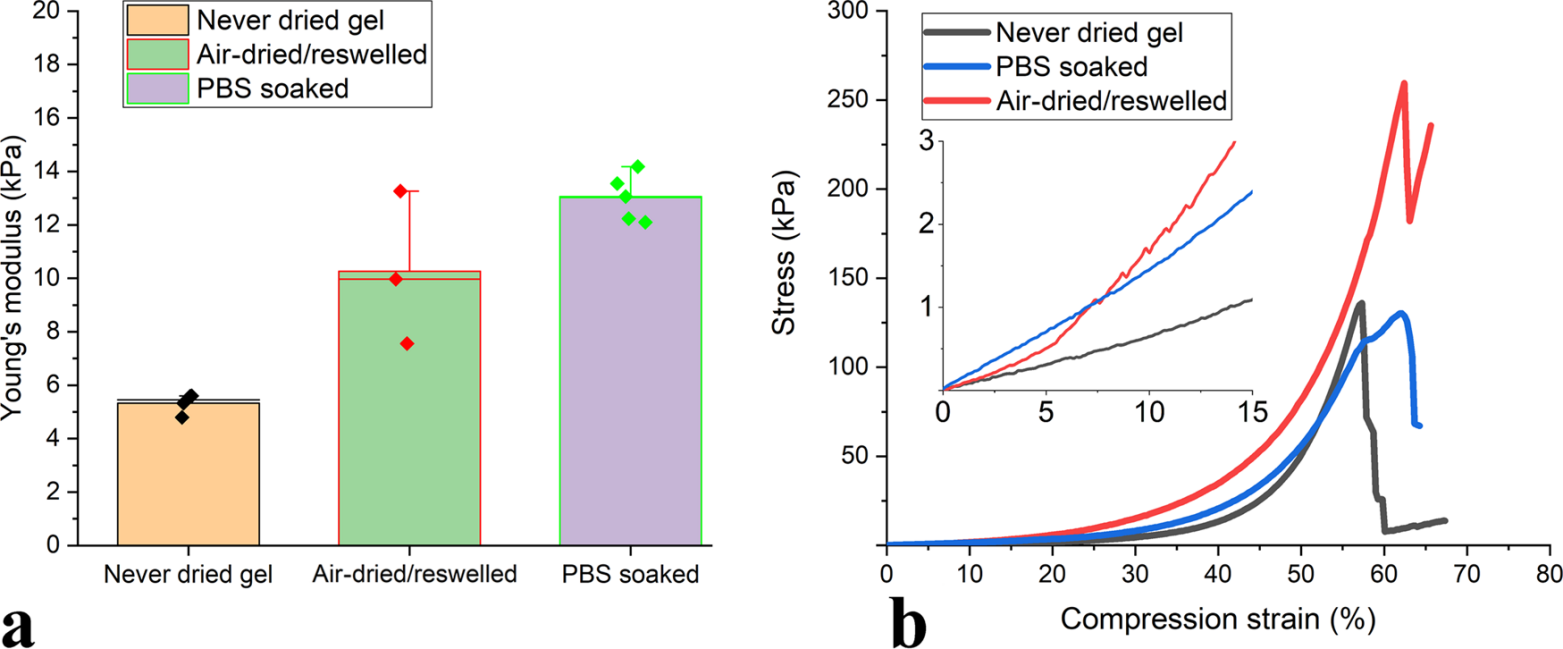
Comparison of the (a) Young’s modulus and (b) stress versus strain results under the compressive load for the 1.1% CNF-MA+1% PAA hydrogels.

Considering the application of the as-prepared CNF-MA+PAA hydrogel in supporting 3D cell culture, the integral stability of the 1.1% CNF-MA+1% PAA hydrogel is also of considerable importance, and therefore, the mechanical properties of the gels were evaluated in compression after soaking in a PBS buffer. After soaking in PBS buffer for 24 h, the shrinkage of the hydrogel sample was observed (around 10% of the linear dimensions), which is expected due to the high ionic strength of the PBS solution, which will lead to the deswelling of the polyelectrolyte gel. At the same time, the Young’s modulus value for the 1.1% CNF-MA+1% PAA hydrogel before (5.3 kPa) was more than doubled after the PBS soaking (13 kPa), as shown in Figure 5a. This is most probably due to the deswelling, which again will lead to a higher concentration of fibrils in the gel and hence a more compact fibrillar network, which will lead to a higher resistance toward compression. Furthermore, the reswollen hydrogel samples prepared by reswelling the air-dried 1.1% CNF-MA+1% PAA films in deionized water resulted in a 2-fold increase in Young’s modulus, i.e., from 5.5 kPa to close to 10.3 kPa. This strongly indicates that the evaporation–reswelling method significantly changes the internal network of the hydrogel and obviously increases the network’s apparent cross-linking density, i.e., cross-links most probably formed by cellulose/cellulose interactions. It is worth noting that there is no significant difference in stress at break for the PAA-cross-linked material before (98.7 kPa) and after (110 kPa) soaking in PBS and that there is a 100% improvement in the stress at break for the air-dried/reswelled material (Figure 5b). However, it must be kept in mind that the reswollen sample is measured in deionized water and not in PBS, and this might have a significant effect on the mechanical properties, since the polyelectrolyte gel will be preloaded due to the internal osmotic pressure.

### Cytotoxicity and Proliferation Tests of Fibroblasts and Cancer Cells on the CNF-MA+PAA Hydrogel

**Figure 6:**
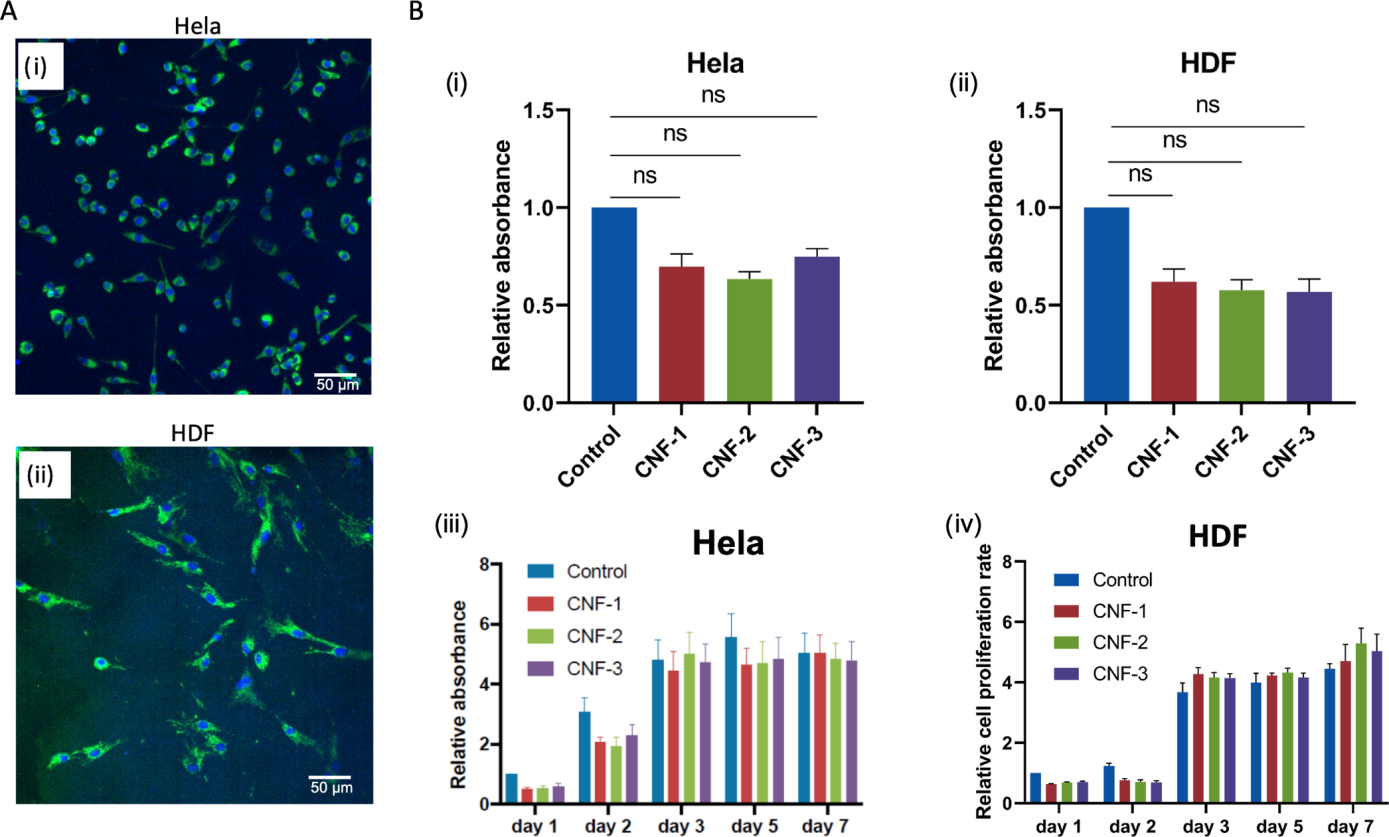
Representative confocal images of the cells were recorded after 48 h of incubation for HeLa and HDF in the 24-well with the glass-bottom plates precoated with different CNF-MA hydrogels (CNF-3). The cell morphology was shown by actin staining (Phalloidin, green), and nuclei were counterstained by DAPI (blue). Scale bar: 50 μm. Cell survival rates and cytotoxicity were carried out for (A.i) HeLa and (A.ii) HDF cultured on the plates precoated with different CNF-MA hydrogels with a density of 5000 cells/well. Cell proliferation rates on the plates precoated with different CNF-MA hydrogels were carried out for (B.i, iii) HeLa and (B.ii, iv) HDF using the CCK-8 assay. Cells were incubated for 1, 2, 3, 5, and 7 days. Bar = mean ± STDEV. In the figures, control: cell culture plastic; CNF-1, -2, and -3 are three types of CNF-MA+PAA hydrogels cross-linked from 1.1% CNF-MA and acrylamide of varied concentrations of 0.25, 0.5, and 1%, respectively.

The cytocompatibility of the cross-linked CNF-MA+PAA hydrogels as a cell culture matrix was preliminarily evaluated with HeLa and HDFs cells. With the cell culture plastic as control, three types of CNF-MA+PAA hydrogels (cross-linked from 1.1% CNF-MA and acrylamide of varied concentrations of 0.25, 0.5, and 1%) were compared in terms of cytotoxicity and cell proliferation behaviors. In the culture of HeLa (Figure 6B.i) and HDF (Figure 6B.ii) cells, all three groups of CNF-MA+PAA hydrogels demonstrated satisfactory cell viability compared to the culture on the two-dimensional (2D) reference (2D Mock). The confocal imaging analysis of the fixed matrices after 48 h incubation also indicated that all of the hydrogel matrices supported both types of cells to grow on the matrix surface, as indicated by the intense distribution of cells stained with two markers: the actin cytoskeleton protein marker Phalloidin and the nuclear marker DAPI (Figures 6 and S14). The classic elongated and widespread morphology of both HDF cells and Hela cells further demonstrates the good cytocompatibility of both investigated cell lines growing on the hydrogel matrices.

Moreover, the CNF-MA+PAA hydrogels still supported cell proliferation after longer incubation periods. The cell proliferation was quantitatively evaluated after day 1 (D1), D2, D3, D5, and D7, as shown in Figure 6B.iii for HeLa cells and in Figure 6B.iv for HDF. At D1 and D2, the cell growth was slightly inhibited in comparison to the 2D Mock control. After 72 h, both HeLa cells and HDF cells recovered high proliferation speed, and the proliferation rate showed no significant deviation from the 2D Mock control. Meanwhile, there was no difference in the cell proliferation between the three groups of CNF-MA+PAA hydrogels, which indicates that the content of polyacrylamide in the hydrogels has a minor influence on the gel’s compatibility in supporting cell proliferation or survival. When studying mechanotransducive cell behaviors, the PAA-based hydrogels with a predesignated stiffness gradient have been widely used as in vitro cell culture platforms to study cellular behavior in response to ECM elasticity. As the PAA hydrogels lack cell adhesive domains, they additionally require a “gel-activating” step with functionalization of Sulfo-SANPAH (sulfosuccinimidyl 6-(4′-azido-2′-nitrophenylamino)hexanoate) to subsequently covalently attach the ECM protein, such as fibronectin, laminin, or collagen. Featured with a low dosage of monomeric AA in resulting sufficient cross-linking, ease of fabrication, and satisfactory cytocompatibility, the CNF-MA+PAA hydrogels have great potential as a novel matrix system in creating stiffness gradient hydrogels with patterned or moving photomasks for UV photopolymerization.

### Validation of Formulation of CNF-MA and AA as Feedstock Inks in DIW Printing

The flow curve of modified CNF-MA displayed a shear-thinning rheology, thus satisfying extrusion-based DIW printing (Figure S2). The 3D printability and shape fidelity of the developed formulations were evaluated by extrusion-based 3D printing with a pneumatic dispensing system and a conical polyethylene nozzle. The printability was optimized for a nozzle diameter of 250 μm and a printing speed of 600 mm/min. After the extrusion, the scaffolds were cross-linked by the printer’s integrated UV source. The formulations containing fixed amounts of acrylamide (1%) and initiator Irgacure 2959 (0.2%), with changes in the CNF-MA concentration from 0.85 to 1.3%, were evaluated to investigate the printability of the prepared gels. As the printing approach allows cross-linking only after extrusion, the printed object is expected to keep its shape until UV cross-linking is applied. Therefore, only formulations with CNF-MA content higher than 1 wt % were found suitable for printing and UV cross-linking.

**Figure 7:**
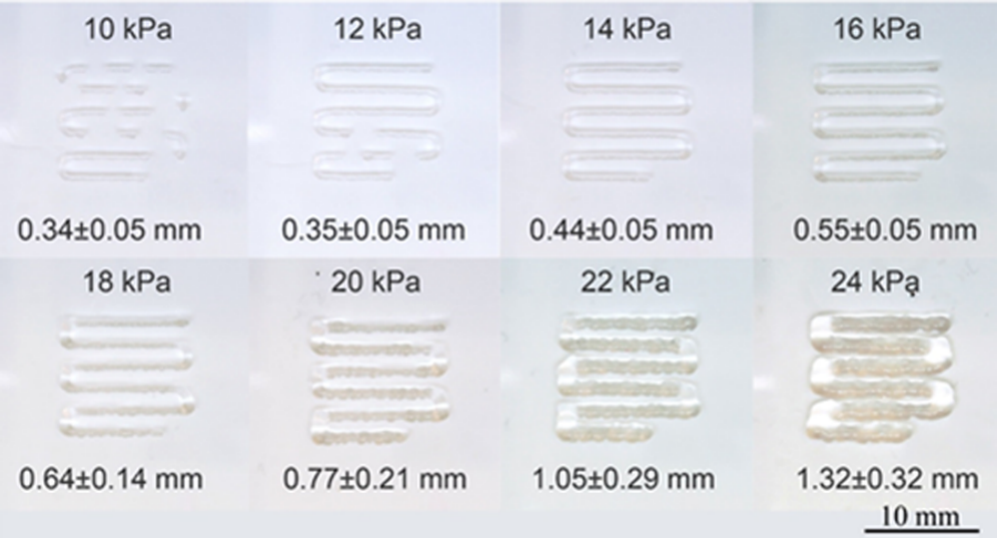
Extrusion of filament using 1.1% CNF-MA+1% acrylamide under varied pressures in a pneumatic dispensing system and a conical polyethylene nozzle (250 μm) with a printing speed of 600 mm/min.

Formulation “Ink-1” with 1.1 wt % CNF-MA was established to best suit the printing conditions. An optimal strut diameter (0.45 mm) for the “Ink-1” formulation was determined for extrusion at 14 kPa pressure (Figure 7a). Moreover, attempts were laid to print complex-shaped objects (Figures S9–S12). To further assess the resolution capacity of the ink formulation, UV cross-linking was used during extrusion. The printed struts were clearly resolved at distances over 1 mm when a 250 μm cylindrical stainless steel nozzle was used together with a printing rate of 240 mm/min and a 100% dispensing rate was set to print the calibration grids with 1–5 mm mesh. In order to determine the maximum number of layers deposited on each other without fusing the gels, a circular grid of 1.5 mm mesh with different numbers of layers was also printed. It was observed that up to 10 layers could be printed on top of each other without significant fusing of gels on the grid. In comparison to the previous studies, where a CNF hydrogel and auxiliary methacrylate components such as gelatine and biopolymer methacrylate were formulated, the current CNF-MA ink showed more suitability in manufacturing relatively soft hydrogel matrix applications.^19,21^ Thus, further development should be explored to extend the stiffness range of the hydrogel by altering the cross-linker and increasing the DS of MA, which is shown to be a challenge while aiming at relatively higher content of biopolymers in the formulation. Moreover, it is worth pointing out that in response to the strong need for precise deposition of biomaterials and cells, a high-resolution construct with a complex structure can be fabricated by vat polymerization that faces a major challenge in developing soft bioresins for cell encapsulation,^37,38^ where the CNF-MA inks likely to have potential and will be investigated.

## Conclusions

We have developed a multistep procedure for the successful preparation of a methacrylated CNF hydrogel with a decent degree of MA and have further assessed the formulation of this photo cross-linkable ink by hydrogel extrusion-based DIW with tuneable mechanical properties in a broad range provided by a combination of CNFs and the cross-linking process. Methacrylation of cellulose can be conducted in several steps, including activation of fibril surfaces within the used fibers by TEMPO-mediated oxidation and surface modification of hydroxyl groups on the fibrils with methacrylic anhydride after solvent exchange from water to DMF. Thereafter, CNF-MA is thus prepared by mechanical defibrillation of cellulose fibers in water suspension using high-pressure homogenization. CNF-MA can then be formulated together with monomeric acrylamide to a hydrogel ink for DIW printing. The mechanical strength of the photocured hydrogel can be tuned in a broad range by altering the amount of monomer content. The resulting hydrogel is superhigh transparent and can reswell in water and expand to all directions to restore its original dimensions after being air-dried, even with further enhanced mechanical properties for two drying cycles, ascribed to the highly cross-linked CNF/polymer network. The satisfactory cytocompatibility assessed in the culture of HeLa cancer cell line and human dermal fibroblasts and the good printability of the hydrogel ensure potential promising applications of CNF-MA in 3D printing and medical applications.

## Supporting Information Available

Additional experimental details and methods; ^1^H NMR spectra for CNF-MA pulp with 0.91 and 1.20 mmol/g charge density; flow sweep of the formulation; DLS data for nonmodified CNF and CNF-MA; BET surface area analysis results; ^1^H NMR and HP-SEC chromatograms of the extracted binding polymers (PAA); water uptake kinetics for freeze-dried and air-dried cross-linked material “Ink-1”; photographs of drying/reswelling of the UV-cross-linked “Ink-1” material; width of the strut at different extrusion pressures (Ink-1); parallelepiped 11 mm × 11 mm × 1.3 mm, 3 layers; grid with 2 mm mesh 4 layers, 10 mm × 10 mm; different complex shapes printed using the “Ink-1” formulation; resolution of the printing with the “Ink-1” formulation; and multiple layer print, circular grid 1.5 mm mesh (PDF)
