# Supplementary material for: Photocross-Linkable and Shape-Memory Biomaterial Hydrogel Based on Methacrylated Cellulose Nanofibres
Source: Biomacromolecules. 2023 Aug 1;24(8):3835–45. doi: 10.1021/acs.biomac.3c00476 (PMC10428165; doi:10.1021/acs.biomac.3c00476)
Supplement: Supplementary file 1 — bm3c00476_si_001.pdf [file bm3c00476_si_001.pdf]

Supporting Information

Photocross-Linkable and Shape-Memory Biomaterial Hydrogel Based on Methacrylated Cellulose Nanofibres

Yury Brusentsev,[a] Peiru Yang,[bc] Alistair W. T. King,[d] Fang Cheng,[e] Maria F. Cortes Ruiz,[f,g] John E. Eriksson,[b,c] Ilkka Kilpeläinen,[d] Stefan Willför,[a] Chunlin Xu,*[a] Lars Wågberg[f,g] and Xiaoju Wang*[a]

17 pages, 13 Figures, 2 Tables

[a]Laboratory of Natural Materials Technology, Johan Gadolin Process Chemistry Centre, Åbo Akademi University, Henrikinkatu 2, 20500 Turku, Finland.

[b]Turku Bioscience Centre, University of Turku and Åbo Akademi University, Tykistökatu 6, 20520 Turku, Finland.

[c]Cell Biology, Faculty of Science and Engineering, Åbo Akademi University, Tykistökatu 6, 20520 Turku, Finland.

[d]Chemistry Department, University of Helsinki, Yliopistonkatu 3, 00014 Helsinki, Finland.

[e]School of Pharmaceutical Sciences (Shenzhen), Shenzhen Campus of Sun Yat-sen University, Shenzhen 518107, China.

[f]Department of Fibre and Polymer Technology, Division of Fibre Technology, KTH Royal Institute of Technology, Teknikringen 56-58, 100 44 Stockholm, Sweden.

[g]Department of Fibre and Polymer Technology, Wallenberg Wood Science Centre, KTH Royal Institute of Technology, Teknikringen 56-58, 100 44 Stockholm, Sweden.

*Corresponding author email address: Chunlin.Xu@abo.fi; Xiaoju.Wang@abo.fi

Preparation of the modified CNF hydrogels and DS determination

Preparation of the modified fibres

10 g (dry weight) of the TEMPO oxidized fibres in water (charge density: 0.82, 1.25 and 1.40 mmol/g) was pressed on a filter to remove the main amount of water. Then the material was dispersed in 300 mL of DMF. The suspension was filtered to remove most of the solvents and 150 mL of DMF was added on filter. After mixing and filtration the material was washed 2 more times with 150 mL of DMF. Next the material was transferred to a flask with a magnetic stirrer and 300 mL of DMF was added. Then 10 mL (73 mmol) of triethylamine was added. After proper mixing of the reaction mixture 8.5 mL (56 mmol) of methacrylic anhydride was added dropwise during 15 min. The reaction mixture was left stirring overnight with light protection and then filtered. The modified fibres were washed on a filter with water (5 x 300 mL) and then concentrated to ≈10% of the dry weight and stored in cold with light protection until homogenization.

Modified fibre analysis

The charge was determined for the prepared fibres by conductometric titration following the protocol reported previously.[1] Average of the 3 titrations resulted with the values of: 1) 0.82 ± 0.03 mmol/g for 0.91 mmol/g starting material, 2), 1.07 ± 0.02 mmol/g for 1.25 mmol/g starting material, and 3) 1.18 ± 0.02 mmol/g for 1.40 mmol/g starting material.

The prepared fibres were analysed by NMR spectroscopy. To prepare the NMR sample the material was first washed to remove sodium ions. The modified fibres were first acidified to pH 2.9 with 0.5 M water solution of the methacrylic acid, stirred for 15 min and then filtered. To the solution, was 100 mL 0.1 M tetraethylamine added. The material was washed on filter with deionized water until the conductivity of the filtrate was less than 5 µS/cm^2. Then the material was freeze dried to remove the residues of water. 50 mg of the dry material was dissolved in 1 mL of the electrolyte containing 20% of n-$Bu_4P^+OAc^-$ and 80% of DMSO-d6.[2] The prepared solution was used for the NMR characterization.

To determine the degree of modification the fibres were analysed by proton NMR spectroscopy with diffusion filtering (ledbpgp2s1d pulse program with 0.2 s diffusion time, 90% and 1500 ms gradient pulse). Methacrylic groups were quantified by comparison of integrals of the areas at 1.7-2.0 5.2-5.7 5.8-6.2 ppm (corresponds to CH_3, C=C-H and C=C-H of the methacrylic group respectively) with integrals of the areas 2.9-3.55 3.55-3.95 4.1-4.5 ppm (corresponds to CH-2,3,5,6 CH-4 and CH-1 respectively).

Because of the absence of C6 protons in glucuronic acid units the total integral of CH-2, 3, 4, 5, 6 was calculated for every charge fibre. It was calculated that the integral of CH-2, 3, 4, 5, 6 protons of the oxidized cellulose should be: 1), 5.73 for the material with the charge 0.82 mmol/g, 2), 5.65 for the material with the charge 1.07 mmol/g, and 3), 5.6 for the material with the charge 1.18 mmol/g.

The ^{1}H-NMR spectra for these 3 materials are presented on **Figure S1a-c**. The integral of CH-2, 3, 4, 5, 6 protons were not exactly equal to the calculated values because of low accuracy of phasing and baseline correction of the polymer NMR. Determination of the accuracy of the method requires additional studies and protocol development.

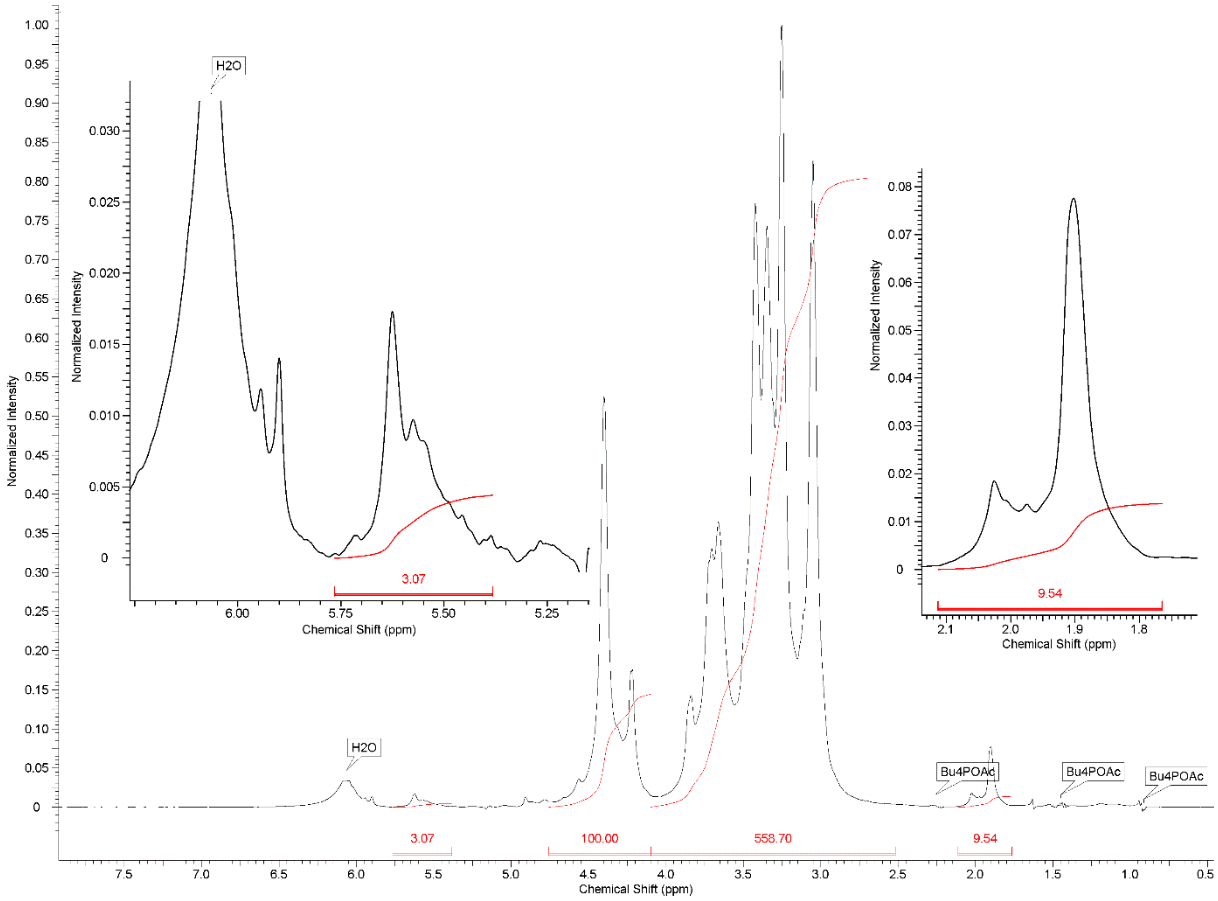

Figure S1a. Diffusion filtered ^{1}H-NMR for methacrylated product from TEMPO oxidized cellulose pulp with 0.91 mmol/g charge density.

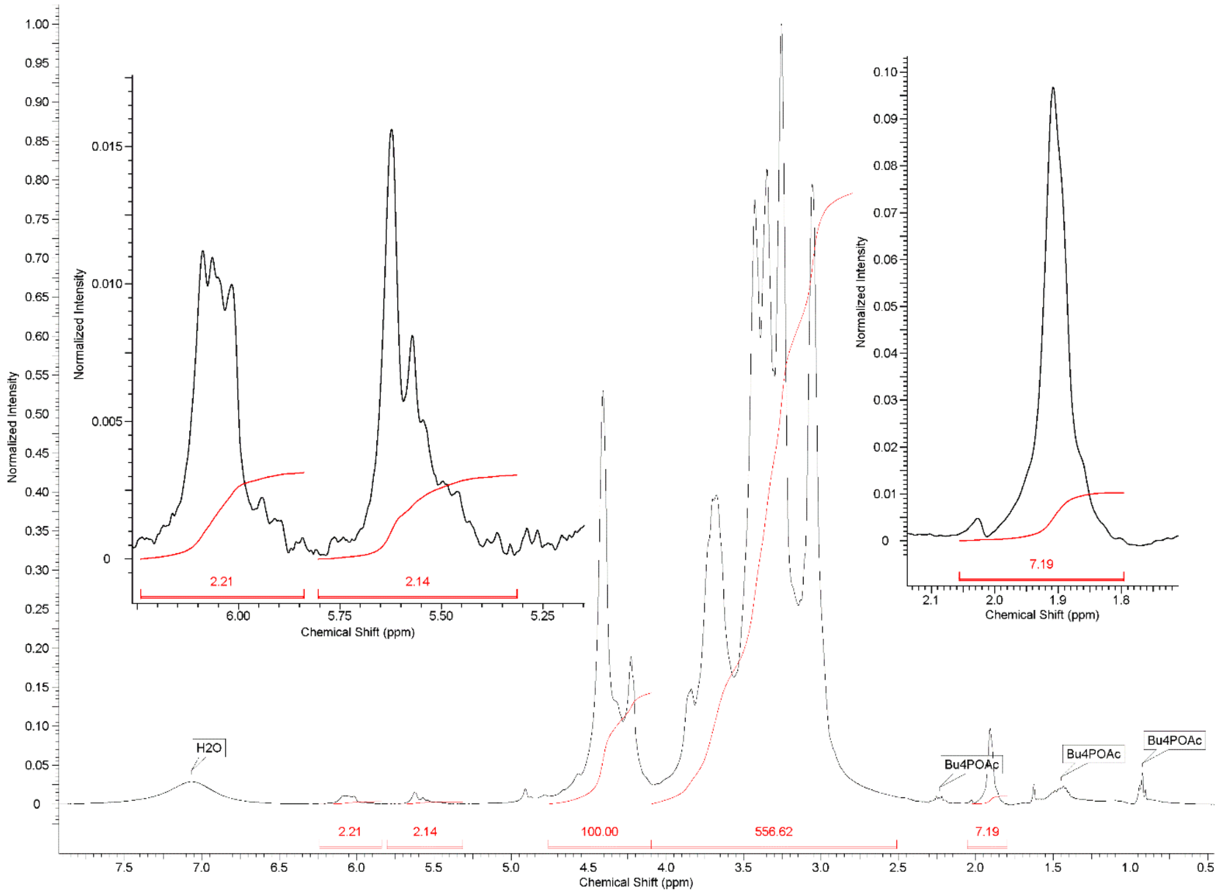

Figure S1b. Diffusion filtered ^{1}H-NMR for methacrylated product from TEMPO oxidized cellulose pulp with 1.2 mmol/g charge density.

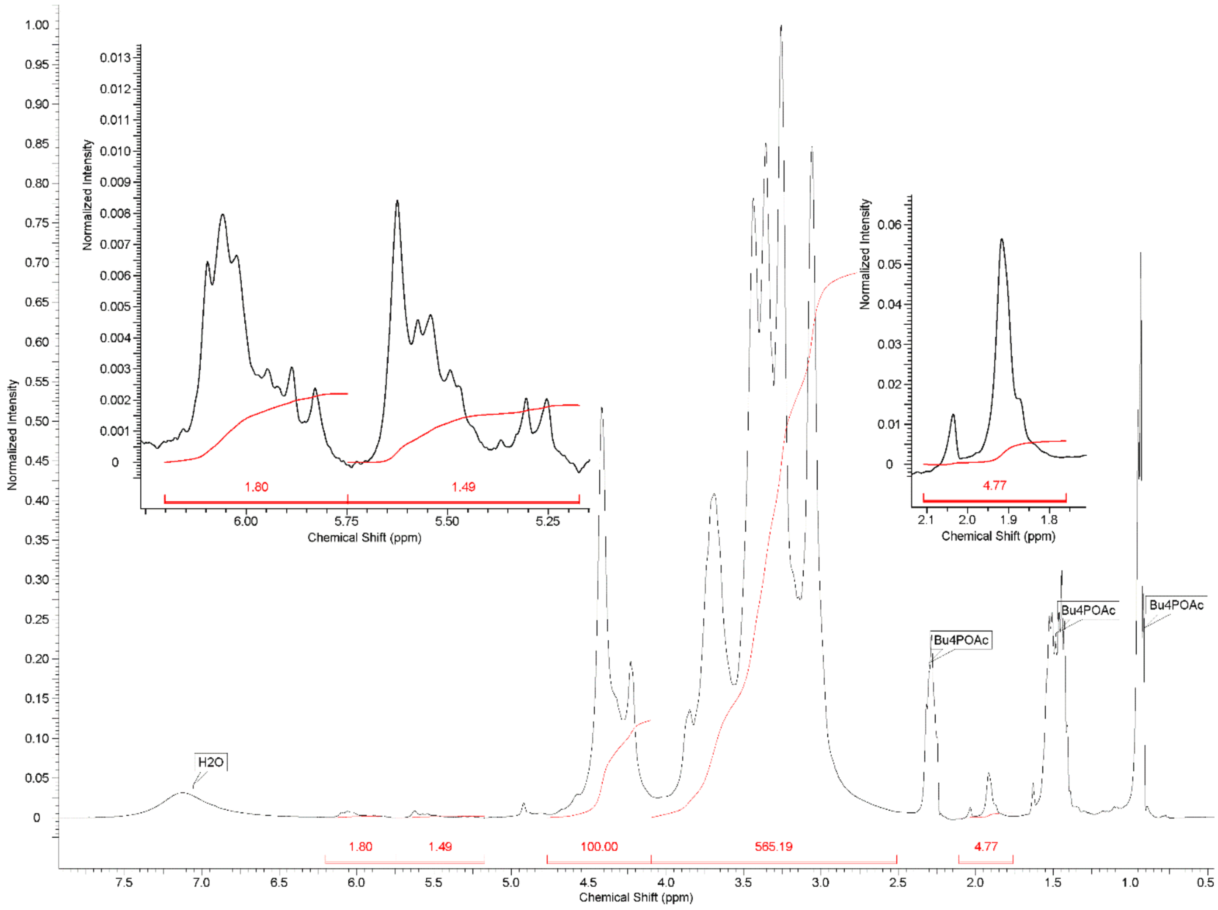

Figure S1c. Diffusion filtered ^{1}H-NMR for products of methacrylated product from TEMPO oxidized cellulose pulp with 1.4 mmol/g charge density.

The amount of the methacrylic groups in these products was determined as:

- DS 0.03 (or ≈0.18) mmol/g for product of modification of 0.91 mmol/g charged cellulose (because of the overlapping with water signal DS was calculated based only on one olefin proton signal)
- DS 0.02 (or ≈0.12 mmol/g) for product of modification of 1.2 mmol/g charged cellulose
- DS 0.014 (or ≈0.08 mmol/g) for product of modification of 1.4 mmol/g charged cellulose

To ensure that acidification and washing did not change the amount of the methacrylic groups on the material the model experiment was performed. 6.5 g of 7.8% TEMPO oxidized fibres (1.2 mmol/g charge, 0.5 g of the dry weight) was dispersed in 100 mL of water and acidified to pH 2 with 0.1 M HCl and stirred for 10 min. The suspension was then filtrated and the material was washed on filter with deionized water until the conductivity of the filtrate was below 5 μS/cm^2. Next, the material was dispersed in water (100 mL) again and 0.35 mL (2.5 mmol) of triethylamine was added to the suspension. The mixture was stirred for 10 min and then filtered. The material was washed with DMF on filter (3 x 20 mL) to remove water and the residue was transferred to a flask with magnetic stirrer and 30 mL of DMF was added. Then 0.5 mL (3.65 mmol) of triethylamine was added. After proper mixing of the reaction mixture 0.43 mL (2.8 mmol) of methacrylic anhydride was added dropwise during 10 min. The reaction mixture was left stirring 4 hours at room temperature with light protection and then filtered. The modified fibres were washed on filter with deionized water until the conductivity of the filtrate was less than 5 μS/cm^2 and then freeze dried. 50 mg of the dry material was dissolved in 1 mL of the electrolyte as it was done for the previous NMR sample. Repeating the NMR analysis as described above gave the same result of the amount of the methacrylic groups of 2.0 mol% of the glucose unit of cellulose material or ≈0.12 mmol/g.
The same NMR analysis for the non-modified TEMPO oxidized fibre (acid washed) gave no signals in the areas corresponding to the methacrylic groups (**Figure S1d**).

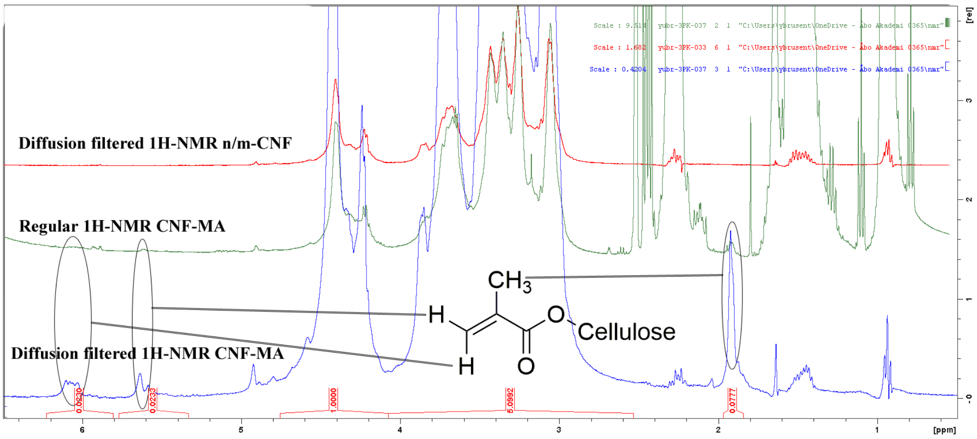

Figure S1d. DS determination of methacrylates by NMR spectroscopy. Comparison of regular ^{1}H-NMR for CNF-MA and diffusion-filtered ^{1}H-NMRs for CNF-MA and n/m-CNF.

The modified fibre with DS 0.02 of methacrylic group was homogenized to the hydrogel (will be called later as CNF-MA) with dry content of 1.4%. Non-modified TEMPO oxidized fibres were homogenized to a hydrogel and used as a reference (will later be called later as n/m-CNF).

To illustrate the shear thinning property of the materials, flow sweep experiment was performed with shear rate changed from 0.001 to 1000 s-1. Different concentration formulations were used for the experiment:

1. CNF-MA 0.85%, acrylamide 1%, Irgacure D-2959 0.2%
2. CNF-MA 1.25%, acrylamide 1%, Irgacure D-2959 0.2%
3. CNF-MA 1.1%, acrylamide 1%, Irgacure D-2959 0.2%

The results are presented on the **Figure S2**:

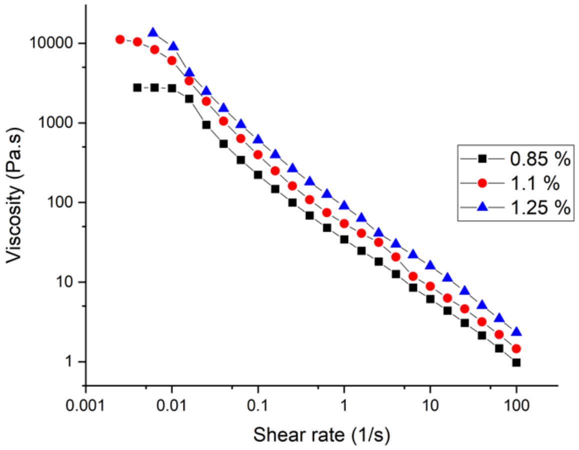

Figure S2. Flow sweep illustrating shear thinning property of CNF-MA of different concentrations with acrylamide 1%, Irgacure D-2959 0.2%.

Dynamic light scattering analysis was performed for 3 samples of 0.05 g/L CNF-MA and 3 samples of 0.05 g/L n/m-CNF at pH 10 to avoid sedimentation. Three repetitions were made for each sample. As a result, the 3 main peaks were observed. Average distributions of the peaks are presented in the **Table S1**:

Table S1. DLS data for non-modified CNF and for CNF-MA.

n/m-CNF	Mode ± SD (nm)	% Pd	% Intensity	% Mass	Polydispersity
Peak 1:	58.77 ± 16.88	27.6	7.7	26	Polydisperse
Peak 2:	342.0 ± 248.0	59	83.5	59.4	Polydisperse

Peak 3:	5560 ± 950.8	22.1	8.8	14.6	Polydisperse

CNF-MA	Mode ± SD (nm)	% Pd	% Intensity	% Mass	Polydispersity
Peak 1:	122.4 ± 45.56	33.1	27.5	14.2	Polydisperse
Peak 2:	615.1 ± 320.5	49.5	68.8	81.5	Polydisperse
Peak 3:	5560 ± 616.7	12.4	3.8	4.3	Polydisperse

Table S2. BET surface area analysis result.

1.1 wt.% CNF-MA	1.1 wt.% n/m-CNF
**Summary Report**	**Summary Report**
Analysis adsorptive: N2	Analysis adsorptive: N2
Analysis bath temp.: 77,455 K	Analysis bath temp.: 77,459 K
Thermal correction: Yes	Thermal correction: Yes
Sample mass: 0,0478 g	Sample mass: 0,0269 g
Ambient free space: 17,1278 cm^3 Measured	Ambient free space: 17,5969 cm^3 Measured
Analysis free space: 56,8557 cm^3	Analysis free space: 58,3057 cm^3
Equilibration interval: 10 s	Equilibration interval: 10 s
Low pressure dose: None	Low pressure dose: None
Sample density: 1,000 g/cm^3	Sample density: 1,000 g/cm^3
Automatic degas: No	Automatic degas: No
**Surface Area**	**Surface Area**
Single point surface area at P/Po = 0,275172089: 440,4807 m^2/g	Single point surface area at P/Po = 0,274347211: 426,2960 m^2/g
BET Surface Area: 455,9822 m^2/g	BET Surface Area: 441,4754 m^2/g
**Pore Volume**	**Pore Volume**
Single point adsorption total pore volume of pores less than 20,568 Å diameter at P/Po = 0,199758027: 0,196405 cm^3/g	Single point adsorption total pore volume of pores less than 20,568 Å diameter at P/Po = 0,199758027: 0,190291 cm^3/g
**Pore Size**	**Pore Size**
Adsorption average pore diameter (4V/A by BET): 17,229 Å	Adsorption average pore diameter (4V/A by BET):17,241 Å
Desorption average pore diameter (4V/A by BET): 82,689 Å	Desorption average porediameter(4V/A by BET):122,259 Å
**BET Report**	**BET Report**
BET surface area: 455,9822 ± 1,3076 m^2/g	BET surface area: 441,4754 ± 1,3469 m^2/g
Slope: 0,009458 ± 0,000027 g/cm^3 STP	Slope: 0,009772 ± 0,000030 g/cm^3 STP
Y-intercept: 0,000088 ± 0,000004 g/cm^3 STP	Y-intercept: 0,000087 ± 0,000004 g/cm^3 STP
C: 108,850235	C: 112,808563
Qm: 104,7614 cm^3/g STP	Qm: 101,4285 cm^3/g STP
Correlation coefficient: 0,9998812	Correlation coefficient: 0,9998610
Molecular cross-sectional area: 0,1620 nm^2	Molecular cross-sectional area: 0,1620 nm^2
**t-Plot Report**	**t-Plot Report**
Micropore volume: 0,007486 cm^3/g	Micropore volume: 0,009167 cm^3/g
Micropore area: 23,3838 m^2/g	Micropore area: 26,5703 m^2/g
External surface area: 432,5984 m^2/g	External surface area: 414,9051 m^2/g
Slope: 27,885094 ± 0,143204 $cm^3/g·Å$ STP	Slope: 26,744002 ± 0,150266 $cm^3/g·Å$ STP
Y-intercept: 4,825673 ± 0,578342 cm^3/g STP	Y-intercept: 5,908947 ± 0,608330 cm^3/g STP
Correlation coefficient: 0,999670	Correlation coefficient: 0,999590

Surface area correction factor: 1,000	Surface area correction factor: 1,000
Density conversion factor: 0,0015514	Density conversion factor: 0,0015514
Total surface area (BET): 455,9822 m²/g	Total surface area (BET): 441,4754 m²/g
Thickness range: 3,5000 to 5,0000 Å	Thickness range: 3,5000 to 5,0000 Å
Thickness equation: Harkins and Jura	Thickness equation: Harkins and Jura
**Thickness Curve**	**Thickness Curve**
t = [13.99 / (0.034 - log(P/Po))] ^ 0.5	t = [13.99 / (0.034 - log(P/Po))] ^ 0.5
**BJH Adsorption Pore Distribution Report**	**BJH Adsorption Pore Distribution Report**
Faas Correction	Faas Correction
Harkins and Jura	Harkins and Jura
t = [13.99 / (0.034 - log(P/Po))] ^ 0.5	t = [13.99 / (0.034 - log(P/Po))] ^ 0.5
Diameter range: 17,000 to 3 000,000 Å	Diameter range: 17,000 to 3 000,000 Å
Adsorbate property factor: 9,53000 Å	Adsorbate property factor: 9,53000 Å
Density conversion factor: 0,0015514	Density conversion factor: 0,0015514
Fraction of pores open at both ends: 0,00	Fraction of pores open at both ends: 0,00

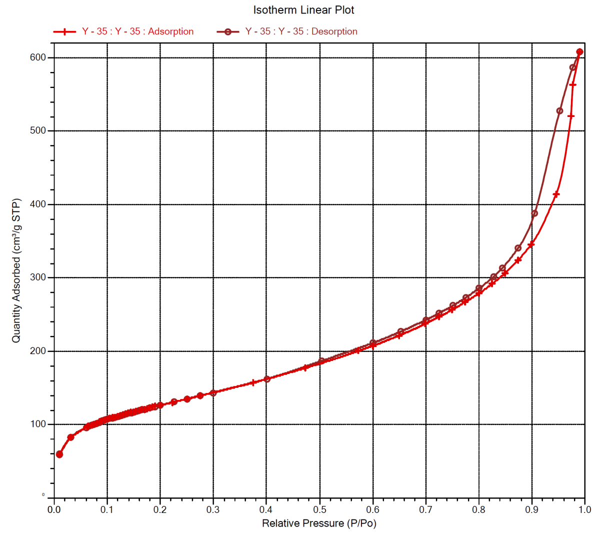

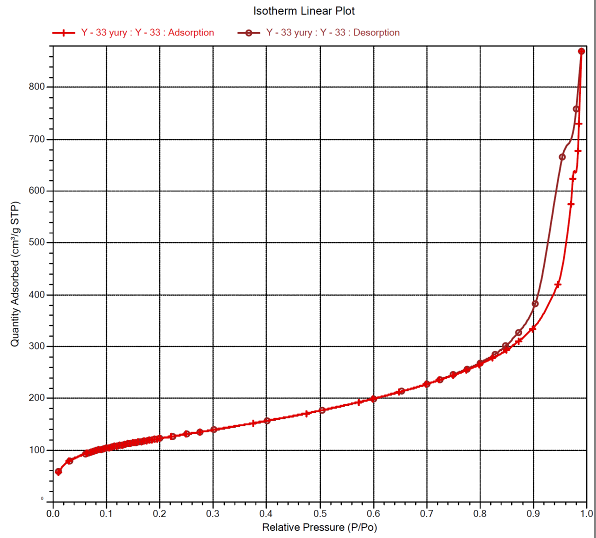

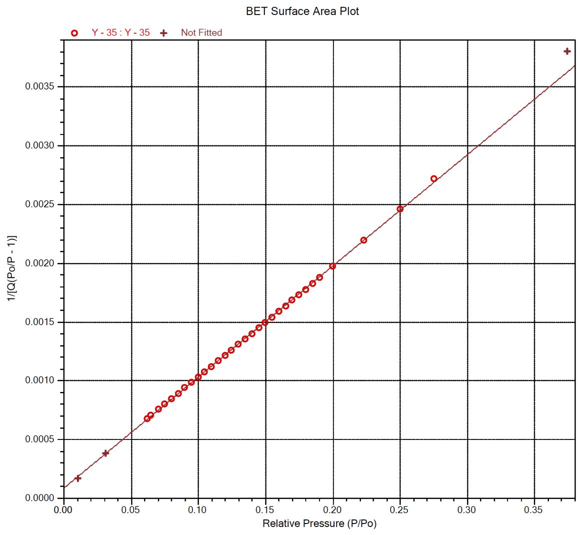

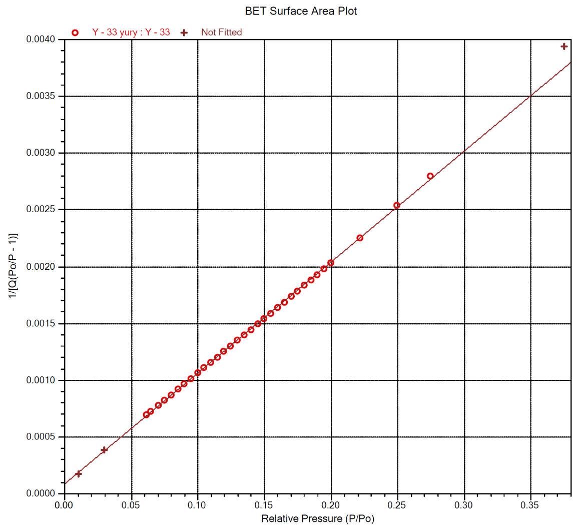

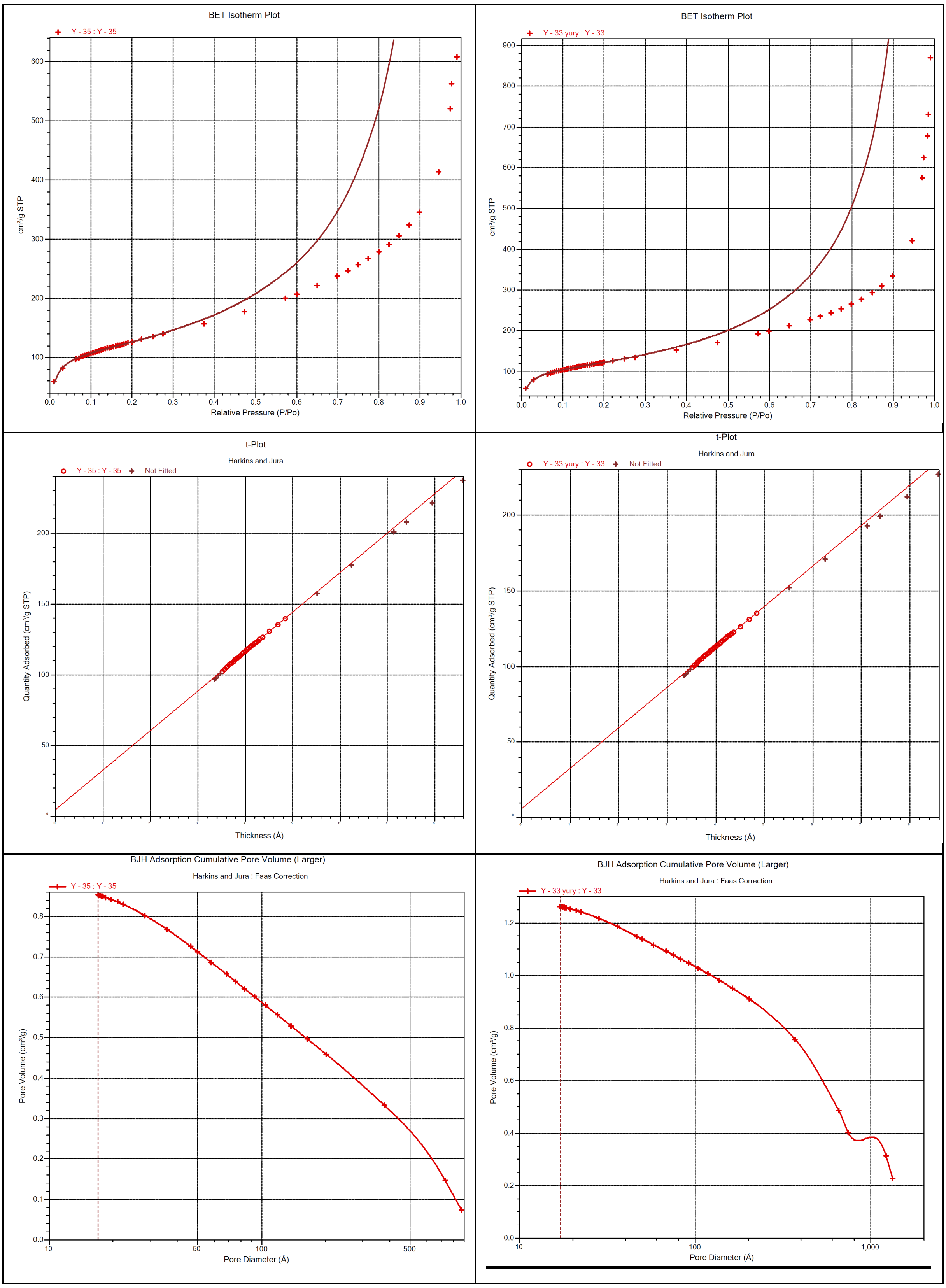
BET Isotherm Plot
Y - 35 : Y - 35
cm³/g STP
Relative Pressure (P/Po)
BET Isotherm Plot
Y - 33 yury : Y - 33
cm³/g STP
Relative Pressure (P/Po)
t-Plot
Harkins and Jura
Y - 35 : Y - 35
Not Fitted
Quantity Adsorbed (cm³/g STP)
Thickness (Å)
t-Plot
Harkins and Jura
Y - 33 yury : Y - 33
Not Fitted
Quantity Adsorbed (cm³/g STP)
Thickness (Å)
BJH Adsorption Cumulative Pore Volume (Larger)
Harkins and Jura : Faas Correction
Y - 35 : Y - 35
Pore Volume (cm³/g)
Pore Diameter (Å)
BJH Adsorption Cumulative Pore Volume (Larger)
Harkins and Jura : Faas Correction
Y - 33 yury : Y - 33
Pore Volume (cm³/g)
Pore Diameter (Å)

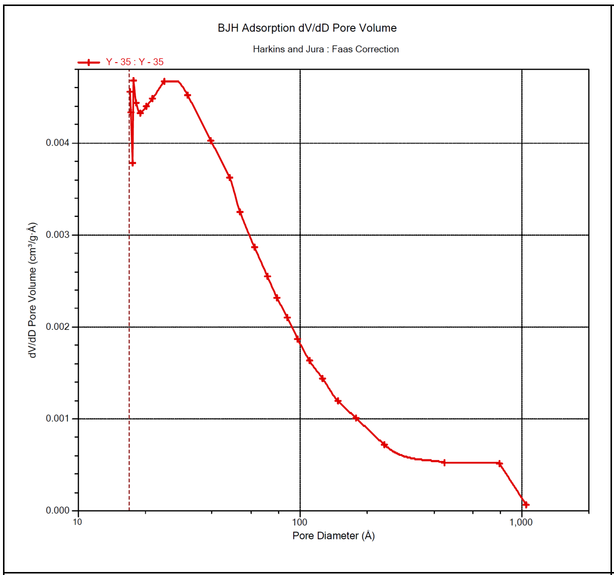
BJH Adsorption dV/dD Pore Volume
Harkins and Jura : Faas Correction
Y - 35 : Y - 35
dV/dD Pore Volume (cm³/g·Å)
0.000
0.001
0.002
0.003
0.004
10
100
1,000
Pore Diameter (Å)

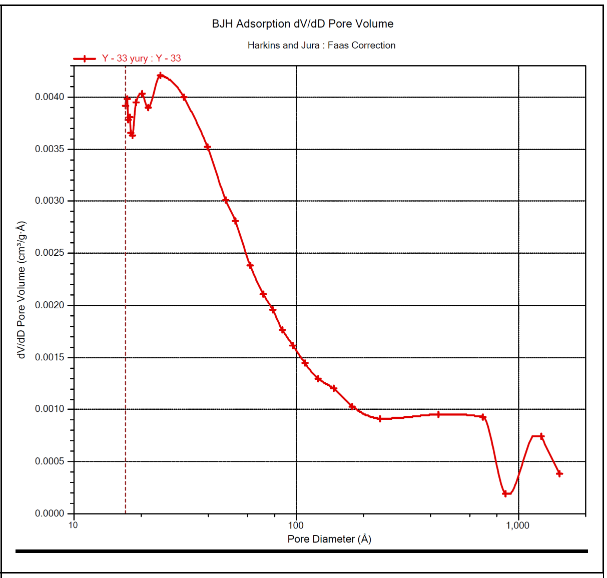
BJH Adsorption dV/dD Pore Volume
Harkins and Jura : Faas Correction
Y - 33 yury : Y - 33
dV/dD Pore Volume (cm³/g·Å)
0.0000
0.0005
0.0010
0.0015
0.0020
0.0025
0.0030
0.0035
0.0040
10
100
1,000
Pore Diameter (Å)

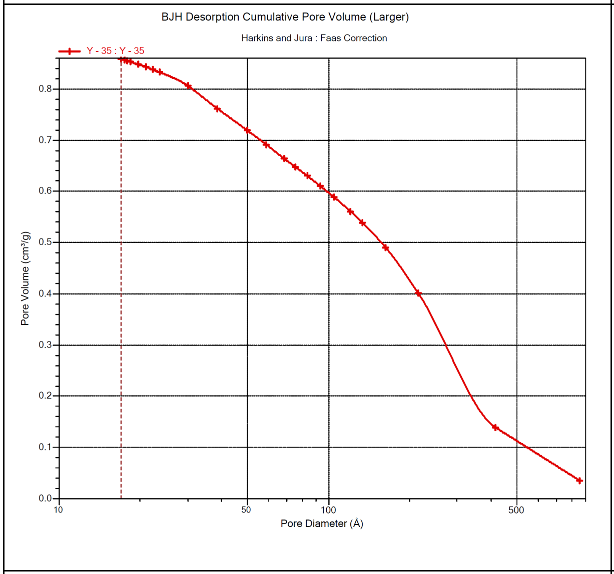
BJH Desorption Cumulative Pore Volume (Larger)
Harkins and Jura : Faas Correction
Y - 35 : Y - 35
Pore Volume (cm³/g)
0.0
0.1
0.2
0.3
0.4
0.5
0.6
0.7
0.8
10
50
100
500
Pore Diameter (Å)

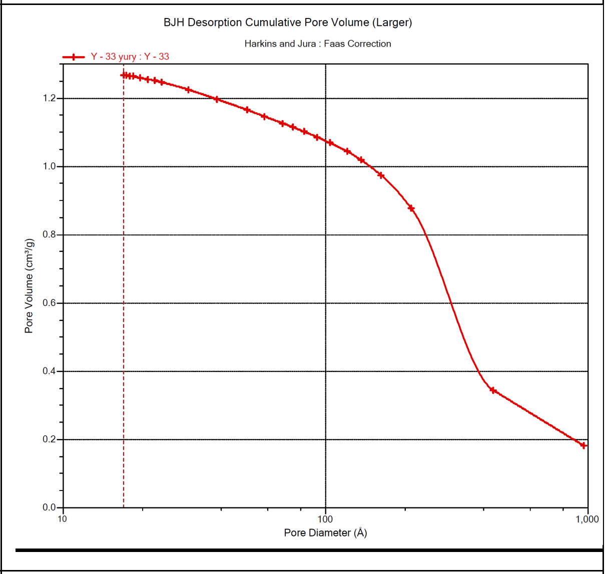
BJH Desorption Cumulative Pore Volume (Larger)
Harkins and Jura : Faas Correction
Y - 33 yury : Y - 33
Pore Volume (cm³/g)
0.0
0.2
0.4
0.6
0.8
1.0
1.2
10
100
1,000
Pore Diameter (Å)

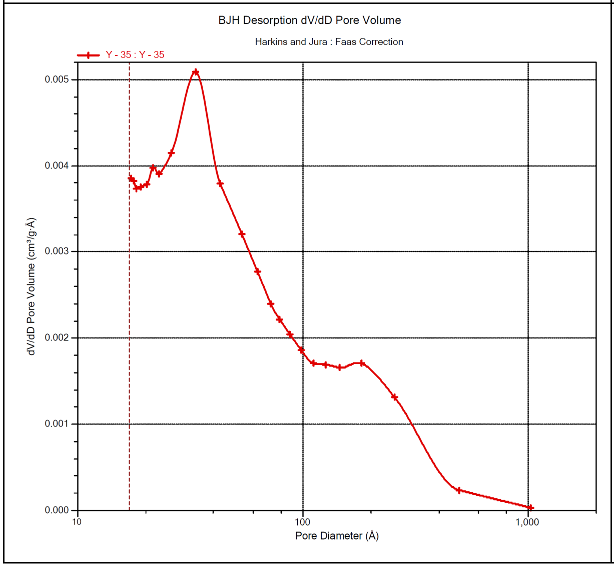
BJH Desorption dV/dD Pore Volume
Harkins and Jura : Faas Correction
Y - 35 : Y - 35
dV/dD Pore Volume (cm³/g·Å)
0.000
0.001
0.002
0.003
0.004
0.005
10
100
1,000
Pore Diameter (Å)

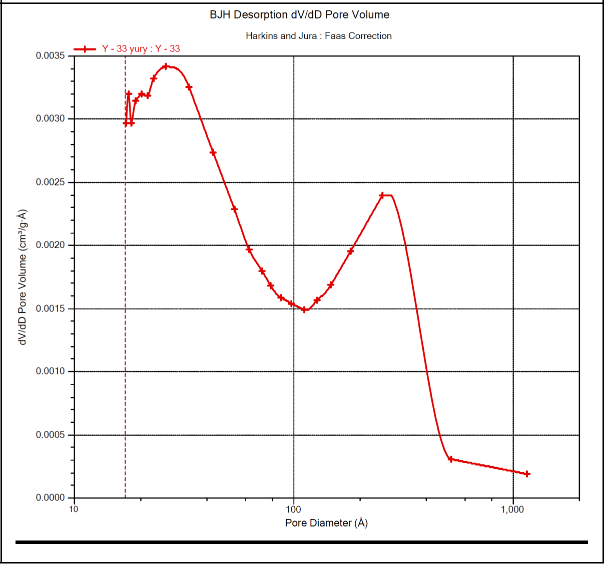
BJH Desorption dV/dD Pore Volume
Harkins and Jura : Faas Correction
Y - 33 yury : Y - 33
dV/dD Pore Volume (cm³/g·Å)
0.0000
0.0005
0.0010
0.0015
0.0020
0.0025
0.0030
0.0035
10
100
1,000
Pore Diameter (Å)

Preparation and characterisation of the UV cross-linkable formulation

Determination of the length of the cross-linker PAA

A 20 g formulation of composed of 1% CNF-MA, 1% AA and 0.2% initiator (Irgacure 2959) was prepared inside of a 50 mL centrifuge tube. After thorough mixing the formulation was crosslinked with a UV light of 365 nm wavelength for at least 30 min. The crosslinked hydrogel was carefully cut into 4 pieces (to decrease out-diffusion path) and submerged into 50 mL of MilliQ water. The hydrogel was allowed to swell in water for 24 hours while changing and collecting the water every 8 hours into a clean round flask. This “wash” water was freeze-dried to purify the unbounded polymer that diffused out of the hydrogel. The dried residue was analysed with NMR and HPLC.

The hydrogel pieces were hydrolysed by placing them in a solution of 25 mL of 0.5 M NaOH and 50 µL of 1 M $NaHCO_3$. The pH of the mixture was around 12.5. The reaction mixture was then covered with parafilm and mixed vigorously for 48 to 72 hours at room temperature until the mixture became a slurry and there were no more pieces of gel remaining. At this point, 1 mL of 12 M HCl was added to neutralize the NaOH and 3 mL more of 1M HCl were added to bring the pH down to 2. It was important to keep the total volume of the mixture low before freeze-drying. The mixture was kept at pH 2 for at least 2 hours. Then the acidified mixture was poured into a dialysis tube with pore size of around 3.5 kDa and dialyzed against MilliQ water for 3 days under constant mixing and a ratio of water to mixture of 100:1. After dialysis, the mixture was filtered using a vacuum filtration set up with a 0.65 µm pore membrane. The residue on the filter was washed another 3-4 times with around 50 mL of MilliQ water. The filter cake formed after filtration should be dried and weight. The filtrate was freeze-dried, weight and analysed for molecular weight and chemical identity using HPLC and NMR.

Initially, the dry content of the material was composed of approximately 200 mg of CNF, 200 mg of AA monomer, and 40 mg of initiator. After extraction and analysis, the unbounded material was found to be 98.9 mg from which 40 mg likely come from the initiator and the resulting 58.9 mg would come from the unbounded PAA. The extracted material after hydrolysis and filtration accounted for 121.4 mg of PAA and the filter cake mostly composed of CNF had a solid content of 215.4 mg. In other words, 29.4% of the polymer was unbounded to the network and 60.7% was cleaved off the hydrogel during hydrolysis. In total 90.1% of the polymer was recovered using this extraction method.

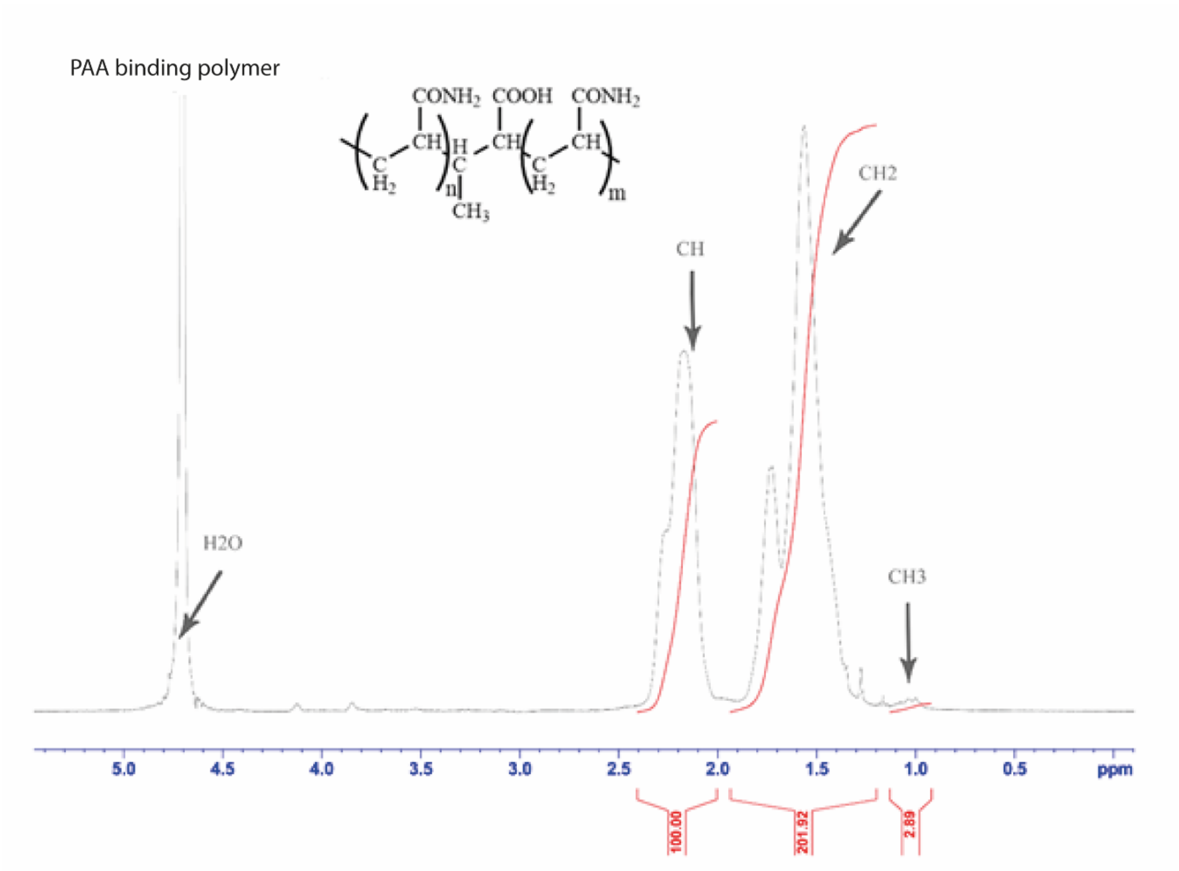

Figure S3. PAA binding polymer ^{1}H-NMR – quantification of methacrylic unit.

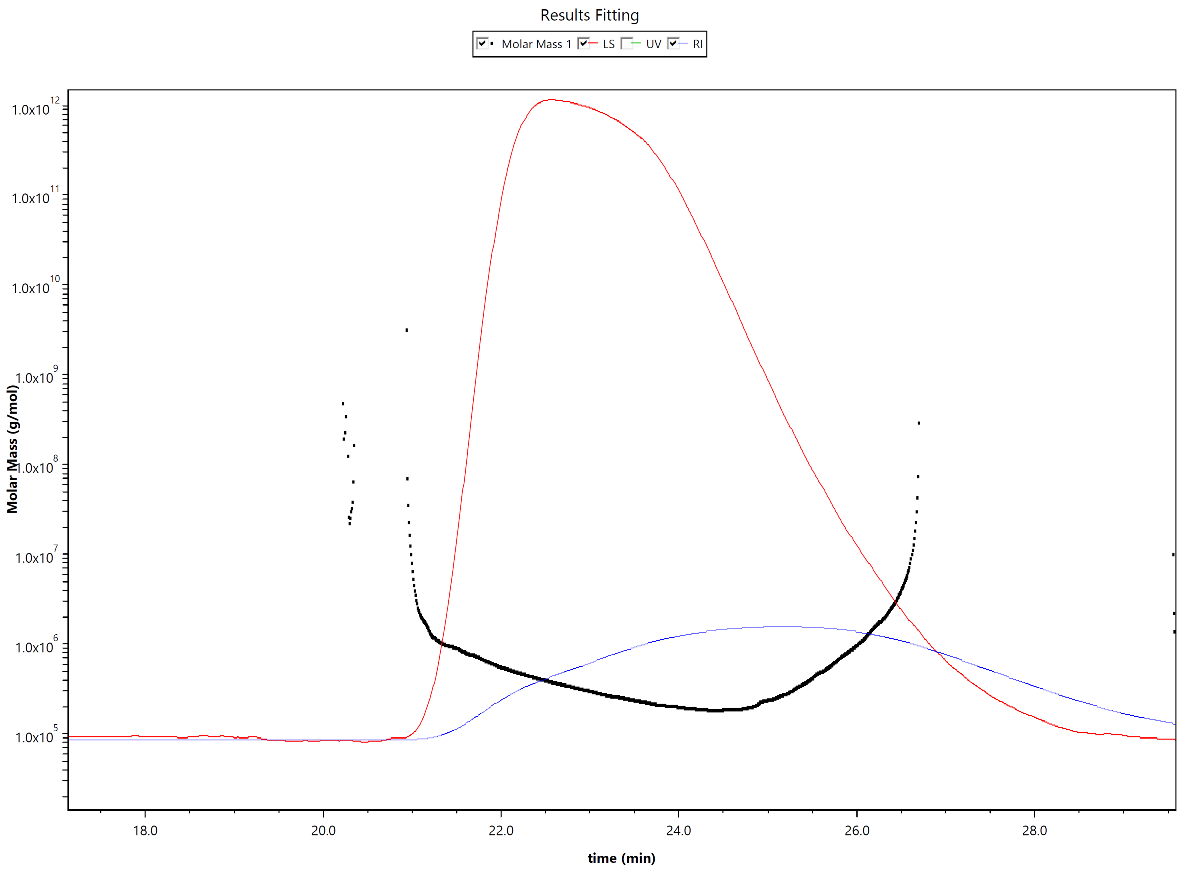

Figure S4. HP-SEC of the extracted binding polymers.

High performance – size exclusion chromatography (HP-SEC) of the polymer for estimation of length of the PAA linker between the fibrils

Average molecular weight of the linking polymer is 300 kDa (polydispersity 1.6) calculated from HP-SEC (**Figure S4**), which corresponds to approximately 4200 acrylamide units. According to the NMR results (signal for the methacrylic methyl group is ~3% of the CH proton of the PAA, Figure S3) the polymer contains around 1% of the methacrylic units, which corresponds to 42 units for average molecule.
Average fibrils with the size 1 µm and thickness 4 nm have molecular weight ~65 MDa and 400000 monosaccharide unit. This means that one fibril with 0.02 DS of the methacrylic groups has around 8000 MA units. This mean that fibrils have around 8 MA groups per nm of their length.
In the scenario when the chain growth occurs along the fibril and the PAA links 2 fibrils together, the linker is attached to around 21 methacrylic groups at the surface of each fibril (~2.6 nm on each fibril). Maximum size of the binding unit would be almost whole polymer length (4200 acrylamide units) – approximately 1200 nm (0.3 nm/acrylamide unit). If there is only one MA group on the fibril incorporated to the PAA link, the average length of the binder between 2 fibrils would be 40 times less ~ 30 nm (around 100 acrylamide units). Real situation must be somewhere in between of these two scenarios. It is also necessary to consider that there should be some length of the PAA at the ends of the polymer, before the first MA group (if the chain growth is not starting from and ending on MA group), which could also decrease maximum length of the linker.

Swelling behaviour

To investigate the prepared material ("Ink 1" - 1.1% CNF, 1% acrylamide and 0.2% Initiator Irgacure 2959, cross-linked with UV) for the swelling ability 2 sets of samples were prepared. Cross-linked gel cylinders were prepared as described for the compression tests. 4 samples were left for 24 h for air-drying at room temperature conditions (22 °C, 25% relative humidity). Weight of the residue was 2.5% (average of 4 samples) of the original gel (dry content 2.3%, the difference of ~10% could be residual moisture content). Three samples were freeze-dried for 18 h to give 2.48% (average of 3 samples) of the original gel (dry content 2.3%, the difference of ~8% could again be residual moisture and an error of the concentration in the prepared gel). The samples were placed then to the deionized water and kinetics of water uptake was determined. The recorded data for the air-dried samples (average of 4) and for freeze-dried samples (average of 3) are presented at **Figure S5**.

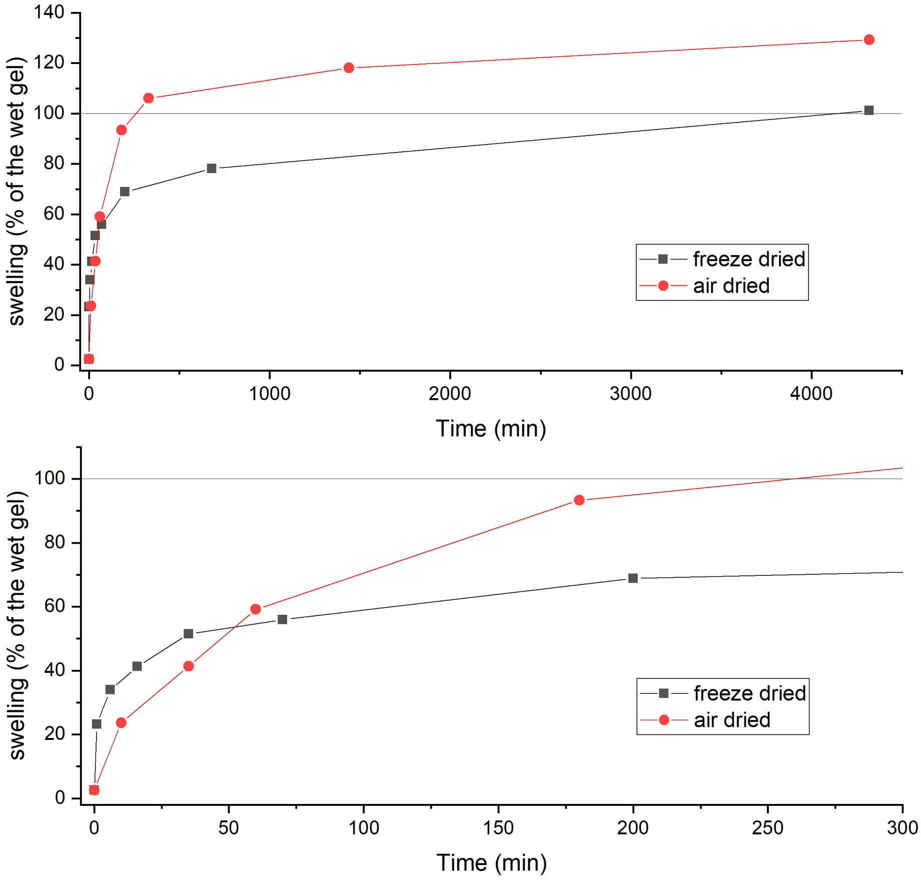

Figure S5. Water uptake kinetics for freeze-dried and air-dried cross-linked material “Ink-1”.

During the experiment it was noticed that freeze-dried samples did not swell as fast to the weight of the original sample. Residues of air were observed inside the freeze-dried samples (see **Figure S6a**). For the air-dried samples it was noticed that after placing in water the sample quickly restore the original shape (see **Figure S6b**).

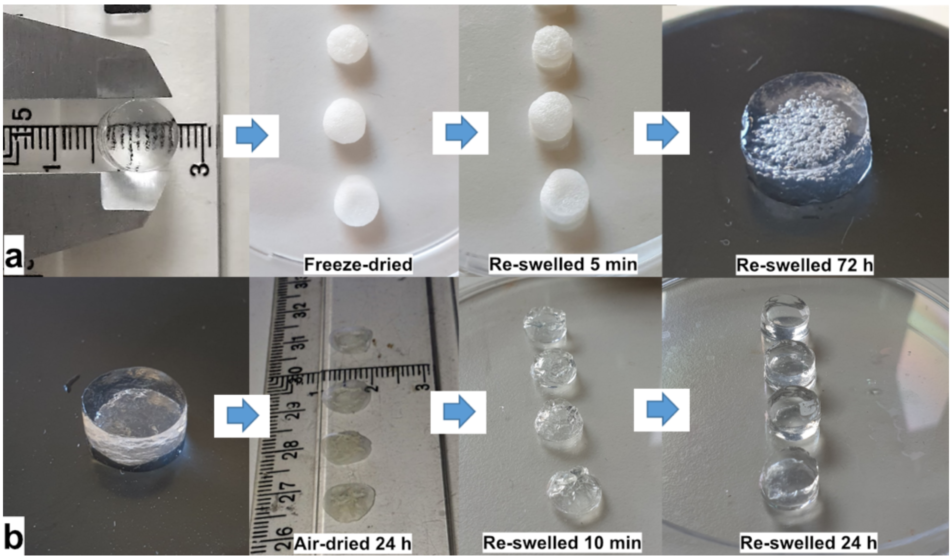

Figure S6. Drying/Re-swelling of the UV-crosslinked “Ink-1” material.

To investigate in more detailed the restoration of the original shape during re-swelling of the air-dried samples several complex-shaped samples were prepared. From the cross-linked “Ink 1” material parallelepiped-shaped sample (1.75 g, 14 mm x 15.5 mm x 8 mm) was cut out. It was left for 35 h for air-drying at room temperature conditions (21 °C, 23% relative humidity). The weight of the shapeless residue was 2.7% of the original gel (dry content 2.4%, the difference of ~15% could again be residual moisture and an error of the concentration in the prepared gel). The residue was placed in water for swelling. After few hours the sample restored the original parallelepiped shape (**Figure S7**).

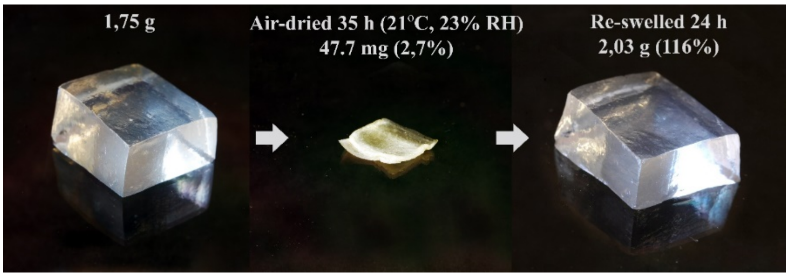

Figure S7. Air-drying – swelling in water of the cross-linked “Ink-1” formulation.

3D printing

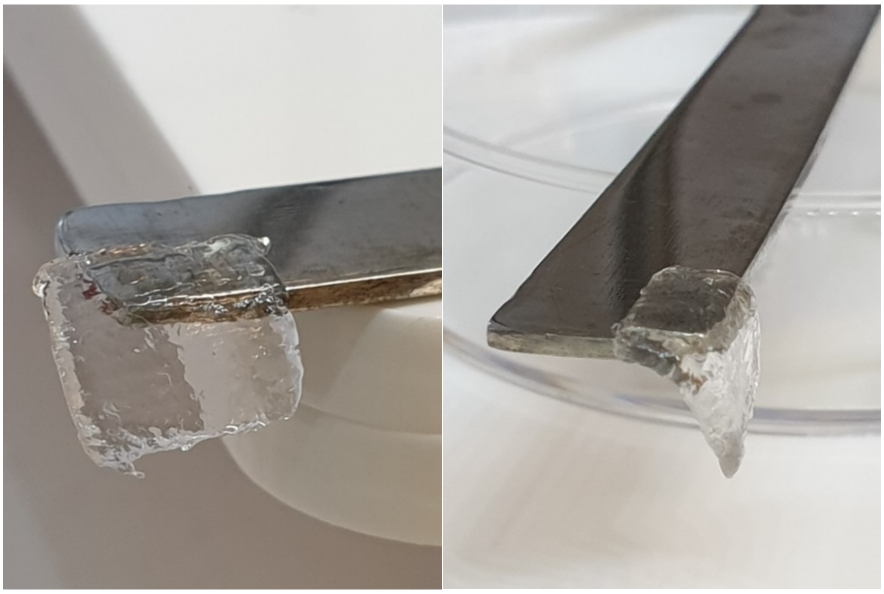

Figure S9. Parallelepiped 11 x 11 x 1.3 mm, 3 layers, **3D printed using pneumatic extrusion**.

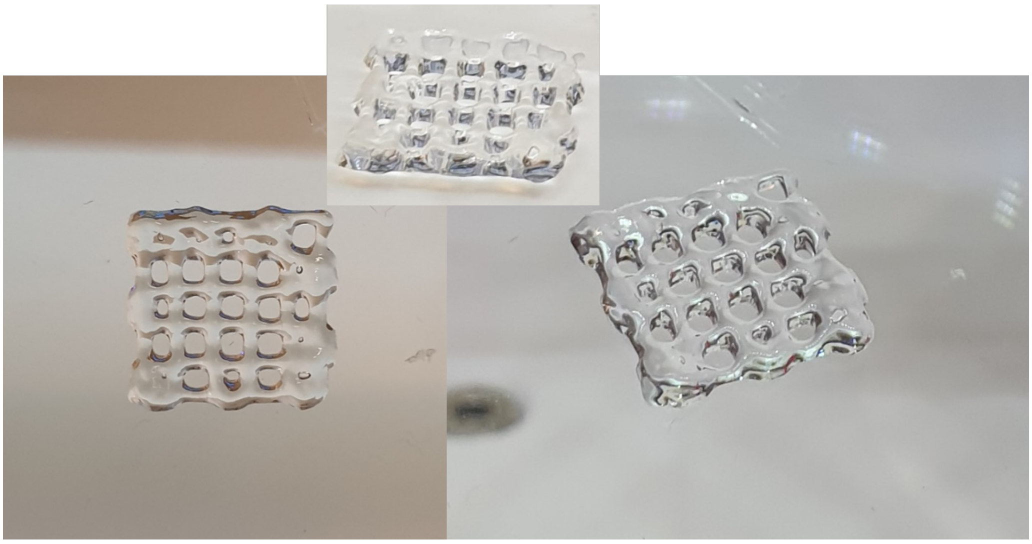

Figure S10. Grid with 2 mm mesh 4 layers, 10x10 mm.

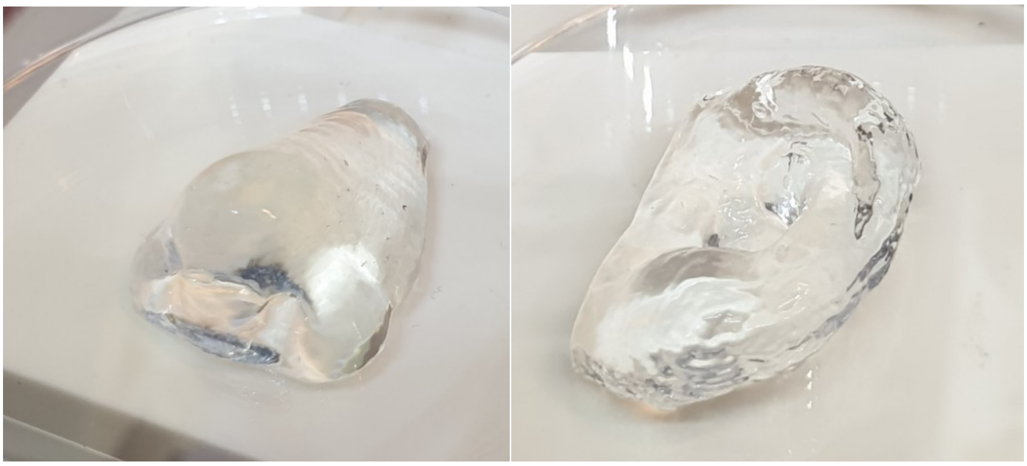

Figure S11. Different complex shapes printed using Ink 1 formulation.

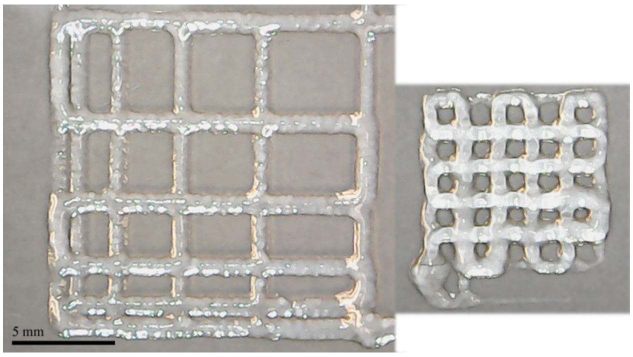

Figure S12. Evaluation of the resolution of 3D printing with ink 1 formulation utilizing screw dispensing system.

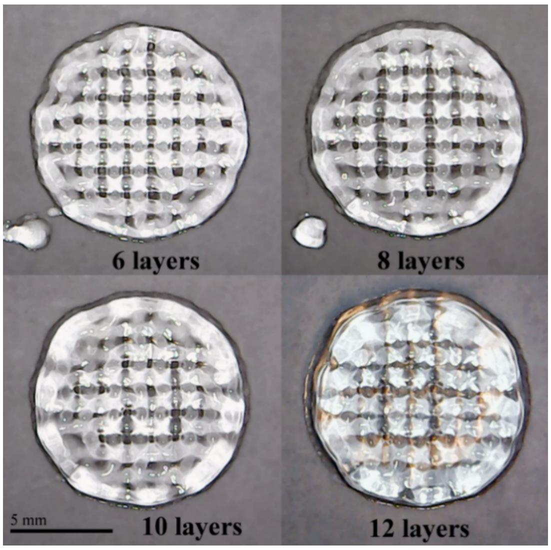

Figure S13. Multiple layer print. Circular grid 1.5 mm mesh.

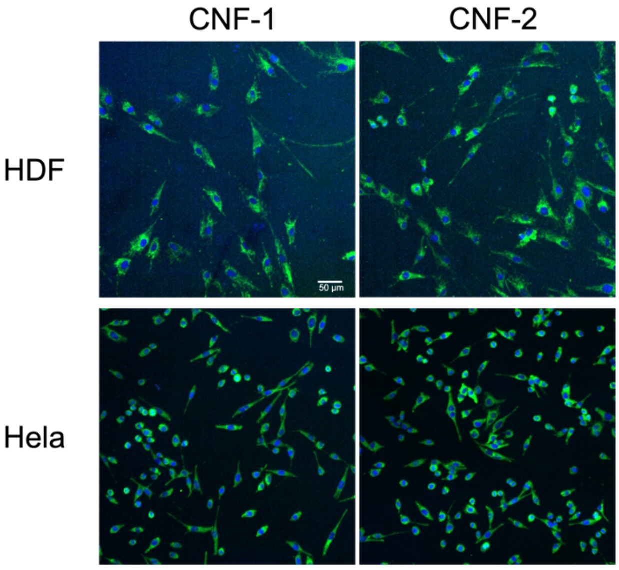

Figure S14. Representative confocal images of the cells were recorded after 48 hours of incubation for HeLa and HDF in the 24-well with the glass-bottom plates pre-coated with CNF-MA hydrogels (CNF-1 and CNF-2). The cell morphology was shown by actin staining (Phalloidin, green) and nuclei were counterstained by DAPI (blue). Scale bar: 50 μm.

References

(1) da Silva Perez, D.; Montanari, S.; Vignon, M. R. TEMPO-Mediated Oxidation of Cellulose III. *Biomacromolecules* **2003**, *4* (5), 1417–1425.

(2) King, A. W. T.; Mäkelä, V.; Kedzior, S. A.; Laaksonen, T.; Partl, G. J.; Heikkinen, S.; Koskela, H.; Heikkinen, H. A.; Holding, A. J.; Cranston, E. D.; Kilpeläinen, I. Liquid-State NMR Analysis of Nanocelluloses. *Biomacromolecules* **2018**, *19* (7), 2708–2720.
